# Adipose tissue senescence is mediated by increased ATP content after a short‐term high‐fat diet exposure

**DOI:** 10.1111/acel.13421

**Published:** 2021-07-18

**Authors:** Maria Pini, Gabor Czibik, Daigo Sawaki, Zaineb Mezdari, Laura Braud, Thaïs Delmont, Raquel Mercedes, Cécile Martel, Nelly Buron, Geneviève Marcelin, Annie Borgne‐Sanchez, Roberta Foresti, Roberto Motterlini, Corneliu Henegar, Geneviève Derumeaux

**Affiliations:** ^1^ Department of Physiology Henri Mondor Hospital, FHU SENEC, INSERM U955 Université Paris‐Est Créteil (UPEC), AP‐HP Créteil France; ^2^ Faculty of Medicine IMRB, INSERM U955 Université Paris‐Est Créteil (UPEC) Créteil France; ^3^ AP‐HP Department of Cardiology Henri Mondor Hospital, FHU SENEC Créteil France; ^4^ Mitologics S.A.S. Université Paris‐Est Créteil (UPEC) Créteil France; ^5^ Sorbonne Universities, INSERM UMR_S 1269, Nutriomics Paris France

**Keywords:** adipose tissue senescence, ATP, bioenergetics, exercise, obesity, xanthine oxidase

## Abstract

In the context of obesity, senescent cells accumulate in white adipose tissue (WAT). The cellular underpinnings of WAT senescence leading to insulin resistance are not fully elucidated. The objective of the current study was to evaluate the presence of WAT senescence early after initiation of high‐fat diet (HFD, 1–10 weeks) in 5‐month‐old male C57BL/6J mice and the potential role of energy metabolism. We first showed that WAT senescence occurred 2 weeks after HFD as evidenced in whole WAT by increased senescence‐associated ß‐galactosidase activity and cyclin‐dependent kinase inhibitor 1A and 2A expression. WAT senescence affected various WAT cell populations, including preadipocytes, adipose tissue progenitors, and immune cells, together with adipocytes. WAT senescence was associated with higher glycolytic and mitochondrial activity leading to enhanced ATP content in HFD‐derived preadipocytes, as compared with chow diet‐derived preadipocytes. One‐month daily exercise, introduced 5 weeks after HFD, was an effective senostatic strategy, since it reversed WAT cellular senescence, while reducing glycolysis and production of ATP. Interestingly, the beneficial effect of exercise was independent of body weight and fat mass loss. We demonstrated that WAT cellular senescence is one of the earliest events occurring after HFD initiation and is intimately linked to the metabolic state of the cells. Our data uncover a critical role for HFD‐induced elevated ATP as a local danger signal inducing WAT senescence. Exercise exerts beneficial effects on adipose tissue bioenergetics in obesity, reversing cellular senescence, and metabolic abnormalities.

## Graphical abstract

High‐fat diet (HFD) induced senescence of various white adipose tissue (WAT) cell types as one of the earliest events preceding mitochondrial dysfunction, inflammation, and fibrosis. In WAT of HFD‐fed mice and preadipocytes derived from HFD‐fed mice, ATP levels were increased. The anti‐senescence effects of exercise were partially dependent on purine catabolism as blocking xanthine oxidase activity increased ATP levels and inhibited exercise's rescue effects in subcutaneous WAT.

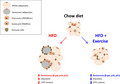

## 1 INTRODUCTION

White adipose tissue (WAT) senescence has emerged as an important contributor to the development of comorbidities associated with obesity (Ahima, 2009; Tchkonia et al., 2010) and aging (Xu et al., 2018). Our group has recently highlighted the impact of WAT senescence on cardiac disorders in the context of aging (Sawaki et al., 2018) and obesity (Ternacle et al., 2017), strengthening evidence that WAT can develop a senescence‐associated secretory phenotype (SASP) that entails the release of various pro‐inflammatory (Campisi, 2005; Coppe et al., 2008; Munoz‐Espin & Serrano, 2014), but also profibrotic proteins (Sawaki et al., 2018), and contribute to spreading senescence to remote organs (Acosta et al., 2013; Tchkonia et al., 2010; Xu et al., 2018). Release of bioactive lipids by WAT may additionally affect the senescence phenotype (Das, 2019). Senescence involves an irreversible arrest of the cell cycle, followed by increased expression of SASP and altered metabolic activity. The accumulation of senescent cells in multiple tissues might drive the aging process and its consequences, such as frailty and the development of chronic diseases (McHugh & Gil, 2018). Therefore, senolytic strategies (Baker et al., 2011, 2016; Xu et al., 2018) are now extensively investigated, while exercise attenuates progression of age‐related disorders (Derumeaux et al., 2008), and also HFD‐induced cardiometabolic disorders (Schafer et al., 2016).

Despite intensive investigation into the role of WAT in metabolic diseases, the timeline and the underlying mechanisms of WAT senescence have not been fully elucidated in the context of HFD. Reports on obesity‐associated WAT senescence focused on the putative role of mitochondrial dysfunction and subsequent increase in reactive oxygen species (ROS) (Lefranc et al., 2019; Lowell & Shulman, 2005). However, recent data pinpointed the impact of ATP released in extracellular space during cell death as part of damage‐associated molecular patterns (DAMP), molecules that induce inflammatory response and are critically involved in the pathogenesis of several diseases (Basisty et al., 2020; Nakahira et al., 2015). Here, we aim to investigate the potential role of mitochondria in obesity‐induced WAT senescence and their modulation by physical exercise with the hypothesis that energetic abundance in caloric overload will lead to overproduction of ATP, contributing to the induction of senescence.

We used a short‐term high‐fat diet (HFD, 1–10 weeks) versus a chow diet (CD) exposure in adult mice (5‐month‐old) to follow the time course of senescence induction in WAT and the impact on glucose homeostasis. We then used physical exercise as a senostatic strategy to investigate whether metabolic alterations were reversed together with WAT senescence in the context of ongoing HFD. Since mitochondria are at the nexus of both metabolic‐ and aging‐associated disorders (Correia‐Melo et al., 2016; Tavallaie et al., 2020), we investigated mitochondrial function in WAT and primary adipocytes.

Here, we demonstrate that WAT senescence: (a) occurred as early as 2 weeks of HFD in both subcutaneous (inguinal, iWAT) and visceral (epididymal, eWAT) WAT preceding the development of chronic systemic inflammation and WAT fibrosis; (b) was associated with increased WAT glycolytic and mitochondrial activity leading to increased local ATP.

We identified a role for increased ATP levels as a causal mechanism in initiating WAT senescence by demonstrating that ATP‐induced preadipocyte senescence *in vitro*, while inhibition of ATP degradation by allopurinol amplified WAT senescence in both *in vitro* and *in vivo* experiments. Furthermore, short‐term exercise confirmed our hypothesis on the role of ATP as a driver of cellular senescence in the context of HFD since it reduced ATP content in iWAT, but not in eWAT and concomitantly prevented HFD‐induced WAT senescence.

## 2 RESULTS

### 2.1 WAT cellular senescence occurs shortly after initiation of HFD

**FIGURE 1:**
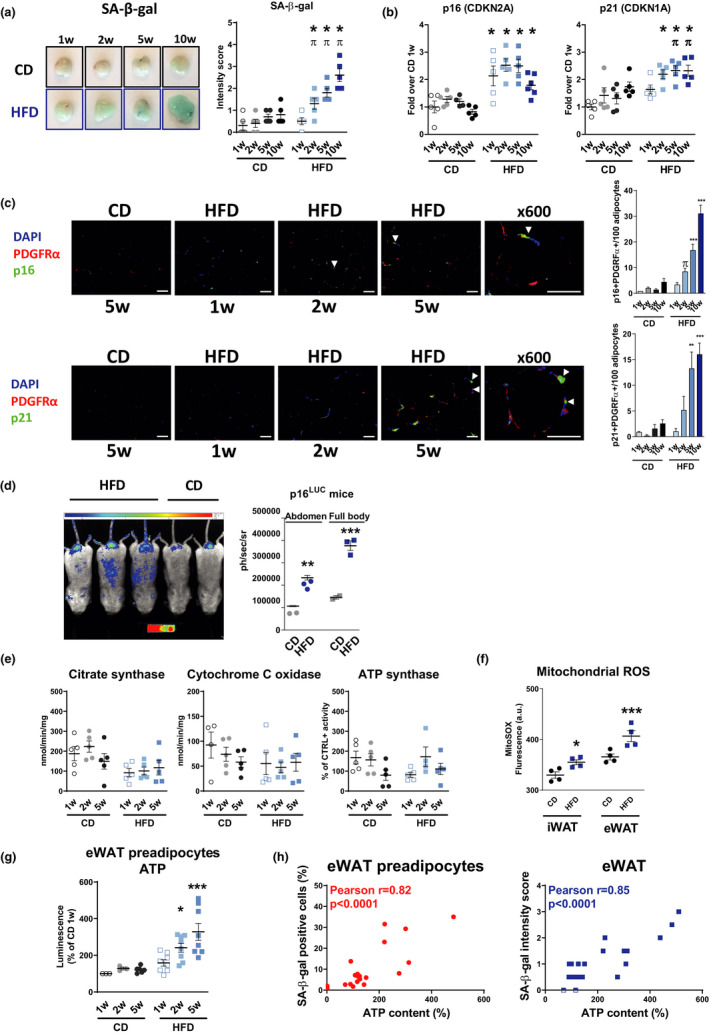
WAT cellular senescence occurs shortly after initiation of HFD. (a–b) Detailed kinetics of epidydimal adipose tissue (eWAT) senescence 1, 2, 5, and 10 weeks after initiation of HFD compared with chow diet (CD) group. Representative images of β‐galactosidase (SA‐β‐gal) activity in eWAT and quantification of relative intensity; n = 5–6 mice/group (a). Gene expression for senescence markers, p16 (Cdkn2a) and p21 (Cdkn1a), in eWAT; *n* = 5 mice/group (b). (c) Representative immunofluorescence images of CD and 1‐, 2‐, and 5‐week HFD eWAT and quantification co‐stained with p16 (green, upper panel) or p21 (green, lower panel) and marker of adipocyte precursor platelet‐derived growth factor receptor alpha, PDGFRα (red) and counterstained with DAPI (blue). Double positive cells are expressed as the mean number of positive cells in percentage of adipocytes; *n* = 3–4 mice/group; magnification ×200, scale bar = 50 μm. For both p16 and p21, in the last panel on the right, a magnified view (600×) of a double positive cell is shown for 5‐week HFD, scale bar = 50 µm. (d) Whole‐body luminescence for individual p16^LUC^ transgenic male mice at 10‐week HFD. Bioluminescence (BLI) is expressed in arbitrary unit (AU; CD *n* = 2, HFD *n* = 3). (e) Citrate synthase activity and activity of respiratory complexes IV (cytochrome C oxidase) and V (ATP synthase) at 1‐, 2‐, and 5‐week time points; *n* = 5 mice/group. (f) Mitochondrial ROS measured in differentiated preadipocytes by MitoSOX fluorescence intensity; *n* = 4 mice/group. (g) Intracellular ATP content in eWAT‐derived preadipocytes 1, 2, and 5 weeks after initiation of HFD compared with CD group; *n* = 3–8 mice/group. (h) SA‐β‐gal activity and ATP content correlated positively in eWAT‐derived preadipocytes (*n* = 25 mice) and in eWAT (*n* = 21 mice). Data are presented either as original images (a, c, d), individual values with (a, b, d–g) or without mean (h) or mean ± SEM (c). Statistical significance was evaluated by one‐way ANOVA followed by Bonferroni correction (a–g) or Pearson correlation (h). *p < 0.05, **p < 0.01, ***p < 0.001 vs. 1‐week CD; π < 0.05 vs. 1‐week HFD

In order to assess the kinetic of WAT senescence after the initiation of HFD, we followed the animals during a 10‐week time course and focused the analysis on eWAT. In parallel with an increase of body weight, fat mass, and leptin expression (Figure S1A), WAT senescence occurred as early as 2 weeks after the initiation of HFD, as demonstrated by a gradual increase in SA‐β‐galactosidase (SA‐β‐Gal) activity (Figure 1a), an upregulation of p16 (cyclin‐dependent inhibitor 2A, Cdkn2a) and p21 (cyclin‐dependent inhibitor 1A, Cdkn1a) (Figure 1b) and a higher frequency of p16‐ (2 weeks) and p21‐ (5 weeks) positive cells in the adipocyte precursor pool (PDGFRα+ cells) (Figure 1c and Figure S1B). SA‐β‐Gal activity also increased in eWAT‐derived primary preadipocytes after 2 weeks of HFD (Figure S1C). Increased WAT senescence was confirmed *in vivo* by a 10‐week HFD intervention in p16^LUC^/+ mice, with higher bioluminescence in the abdominal area, corresponding to the activation of p16INK4a (Figure 1d). Since obesity may compromise mitochondrial homeostasis, we next investigated mitochondrial function to assess its contribution to cellular senescence. During the first five weeks following HFD, we found no evidence of mitochondrial dysfunction based on the assessment of key mitochondrial genes (cytochrome C and Tfam) (Figure S1D), the activity of citrate synthase and of respiratory complexes, such as complex IV (cytochrome C oxidase) and complex V (ATP synthase) (Figure 1e). In addition, we found no evidence for oxidative DNA damage (8‐oxoguanine staining—data not shown), despite a modest increase in mitochondrial ROS (MitoSOX), but not cytosolic ROS (CellROX—not shown) in both iWAT and eWAT HFD‐derived preadipocytes compared with CD preadipocytes (Figure 1f). Remarkably, we found an increase in ATP content in eWAT‐derived preadipocytes as early as 2 weeks (Figure 1g), while ATP levels positively correlated with SA‐β‐Gal activity in both preadipocytes and eWAT (Figure 1h).

### 2.2 HFD induces WAT cellular senescence, independent of systemic inflammation, while exercise prevents it

**FIGURE 2:**
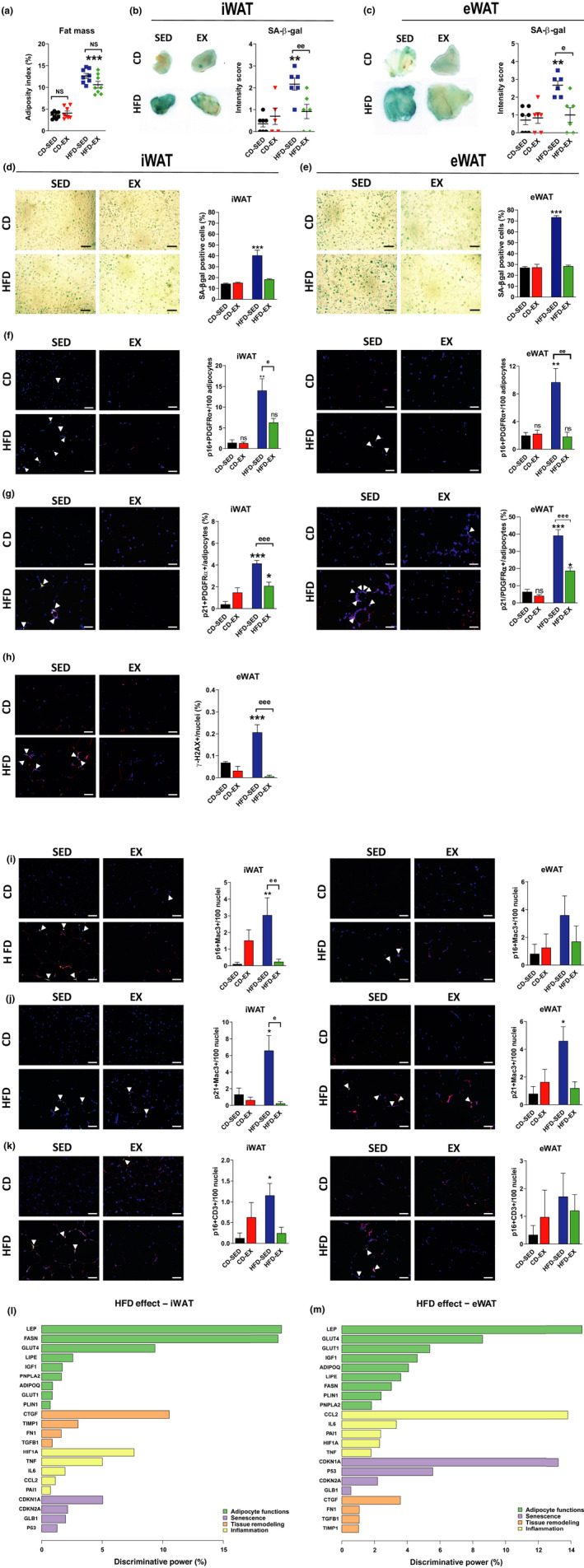
HFD induces WAT cellular senescence, independent of systemic inflammation, while exercise prevents it. (a) Fat mass (adiposity index) of four groups of mice: CD‐sedentary (CD‐SED), CD‐exercise (CD‐EX), HFD‐sedentary (HFD‐SED), HFD‐exercise (HFD‐EX); *n* = 8 mice/group. (b–c) Representative images of SA–β‐gal in iWAT and eWAT and quantification of relative intensity of signal; *n* = 5–7 mice/group. (d–e) Representative images and quantification of SA–β‐gal in preadipocytes derived from iWAT and eWAT expressed in percentage of total cells; *n* = 4 mice/group, magnification ×200, scale bar = 50 μm. (f) Representative images and quantification of co‐localizing p16 (green) and PDGFRα (red) in iWAT and eWAT. Sections were counterstained with DAPI (blue). Double positive cells are expressed as the mean number of positive cells in percentage of adipocytes; *n* = 4–5 mice/group, magnification ×200, scale bar = 50 μm. (g) Representative images and quantification of co‐localizing p21 (green) and PDGFRα (red) in iWAT and eWAT. Sections were counterstained with DAPI (blue). Double positive cells are expressed as the mean number of positive cells in percentage of adipocytes; *n* = 4 mice/group, magnification ×200, scale bar = 50 μm. (h) Representative images and quantification of co‐localizing S139 phosphorylated form of γH2AX (green), double‐stranded DNA break DNA damage marker, and leukocyte marker CD45 (red) counterstained with DAPI (blue) in eWAT. Positive cells are expressed as the mean number of foci in percentage; *n* = 3 mice/group, magnification ×200, scale bar = 50 μm. (i–k) Representative images and quantification of co‐localizing p16 (green) with macrophages, Mac3 (red) (i); p21 (green) with macrophages, Mac3 (red) (j); and p16 (green) with T lymphocytes, CD3 (red) (k) with DAPI (blue) in iWAT and eWAT. Double positive cells are expressed as the mean number of positive cells in percentage of DAPI‐positive nuclei; *n* = 3–5 mice/group, magnification ×200, scale bar = 50 μm. (l–m) The discriminative power of four major functional themes illustrating the HFD impact on WAT transcriptional profile. Transcriptional changes of a panel of selected genes were evaluated separately in iWAT (l) and eWAT (m). Their discriminative power was estimated by iterative testing in a supervised predictive model and is expressed as percentage of their total discriminative power. Data are presented as original images (b–h), individual values plus mean (a–c), or mean ± SEM (d–k). Statistical significance was evaluated by one‐way ANOVA followed by Bonferroni correction (a–k). *p < 0.05; **p < 0.01; ***p < 0.001 for differences due to diet regimen within sedentary and exercise groups (* = diet effect) and ^e^ *p* < 0.05; ^ee^ *p* < 0.01; ^eee^ *p* < 0.001 for differences between sedentary and exercise groups fed the same diet (^e^ = exercise effect)

We then assessed the beneficial effect of exercise (daily swimming) on WAT remodeling. Exercise was initiated after 5 weeks of HFD in comparison with sedentary HFD and CD animals (Figure S2A). To avoid the confounding effect of body weight loss with exercise, for further analysis, we selected mice with similar body weight (Figure S2B) and fat mass (Figure 2a) in each regimen group. As expected, HFD progressively increased body weight (Figure S2B), adiposity (Figure 2a), and adipocyte size (Figure S2C‐D). Exercise reduced adipocyte size in both WAT depots and iWAT weight (Table S1), without altering the food intake (CD‐SED 2.8 ± 1 g/day vs. CD‐EX 3.4 ± 0.6 g/day, *p* = NS and HFD‐SED 3.7 ± 1.0 g/day vs. HFD‐EX 3.7 ± 1.2 g/day, *p* = NS). Although 10 weeks of HFD did not induce WAT fibrosis (Figure S2E‐G), it increased the expression of key profibrotic genes, such as transforming growth factor β1 (Tgfb1), fibronectin (Fn1), and tissue inhibitor of metalloproteinase 1 (Timp1) in eWAT but not in iWAT. Exercise significantly reduced the expression of these profibrotic genes in eWAT of HFD animals (Figure S2H).

HFD increased leptin in both plasma and WAT and reduced adiponectin in plasma (Figure S2I‐J) along with impaired glucose tolerance (GTT) and insulin sensitivity (ITT) (Figure S2K–L). Despite similar body weight, exercise reduced plasma leptin levels in HFD mice, increased plasma adiponectin levels in CD but not in HFD mice, and improved glucose homeostasis only in the HFD group.

Adipogenic function declined early in HFD mice as demonstrated by the downregulation of markers of lipogenesis (fatty acid synthase, Fasn) and lipolysis (perilipin 1, Plin1) in both iWAT and eWAT (Figure S2M). HFD also reduced the expression of insulin‐sensitive glucose transporter (solute carrier family 2 member 4, Slc2a4) and hormone insulin‐like growth factor 1 (Igf1) mRNA in WAT (Figure S2N).

We next focused on the impact of exercise on WAT senescence. While SA‐β‐Gal activity was increased after 10 weeks of HFD in both WAT depots and derived preadipocytes, it was normalized by exercise (Figure 2b–e). HFD increased p21 expression in both iWAT and eWAT, but exercise reduced it only in iWAT (Figure S2O). HFD upregulated p16 and p21 expression in eWAT‐ but not in iWAT‐derived preadipocytes, while exercise normalized it (Figure S2P). Of note, senescence was not influenced by culture conditions prior to differentiation (Figure S2Q). In addition, WAT senescence was associated with adipocyte hypertrophy (Figure S2R). HFD increased the number of crown‐like structures (Figure S2S). Remarkably, WAT senescence occurred without evidence of systemic inflammation as plasmatic levels of pro‐inflammatory factors [C‐C‐Chemokine‐Ligand (CCL2), tumor necrosis factor α (TNFα), interleukin 6 (IL6), interleukin 10 (IL10)] were undetectable. Despite upregulation of Mcp1/Ccl2 in eWAT but not iWAT (Figure S2T), HFD did not significantly increase levels of pro‐inflammatory factors in eWAT secretome (Figure S2U).

Despite the lack of such changes, a 10‐week HFD intervention substantially increased p16+/PDGFRα+ in both iWAT and eWAT (Figure 2f; Figure S2 *Fbis*). Similarly, p21+/PDGFRα+ increased in both iWAT and eWAT (Figure 2g; Figure S2 *Gbis*). Interestingly, exercise blunted the frequency of p16‐ and p21‐positive cells in both WAT depots (Figure 2f–g). The HFD‐induced adipose tissue senescence was further supported by an increase in the S139 phosphorylated form of γH2A.X expression—a marker of double‐stranded DNA break and senescence (Vergoni et al., 2016), which was ameliorated by exercise (Figure 2h; Figure S2 *Hbis*). HFD‐induced p16 and p21 expression in Mac3+ macrophages and p16 in CD3+ lymphocytes in iWAT, which was rescued by exercise. A similar trend was observed in eWAT albeit significant only for p21 in Mac3+ macrophages (Figure 2i–k; Figure S2I‐*Kbis*).

To better discriminate the individual importance of the factors involved in the HFD‐driven alterations of WAT, we applied a supervised predictive model to a panel of transcriptional descriptors selected to illustrate adipocyte function, senescence, inflammation, and tissue remodeling in iWAT (Figure 2l) and eWAT (Figure 2m). Interestingly, the best predictors of HFD‐induced changes in eWAT were leptin and the chemotaxis marker Ccl2, followed closely by the senescence marker p21, while leptin increase was the main change occurring in iWAT.

Altogether, these data reveal that WAT cellular senescence induced by HFD affects multiple cell populations of adipogenic and immune origins in both depots, is rescuable by exercise, and precedes systemic inflammation.

### 2.3 HFD‐induced WAT senescence is associated with increased adipocyte bioenergetics

**FIGURE 3:**
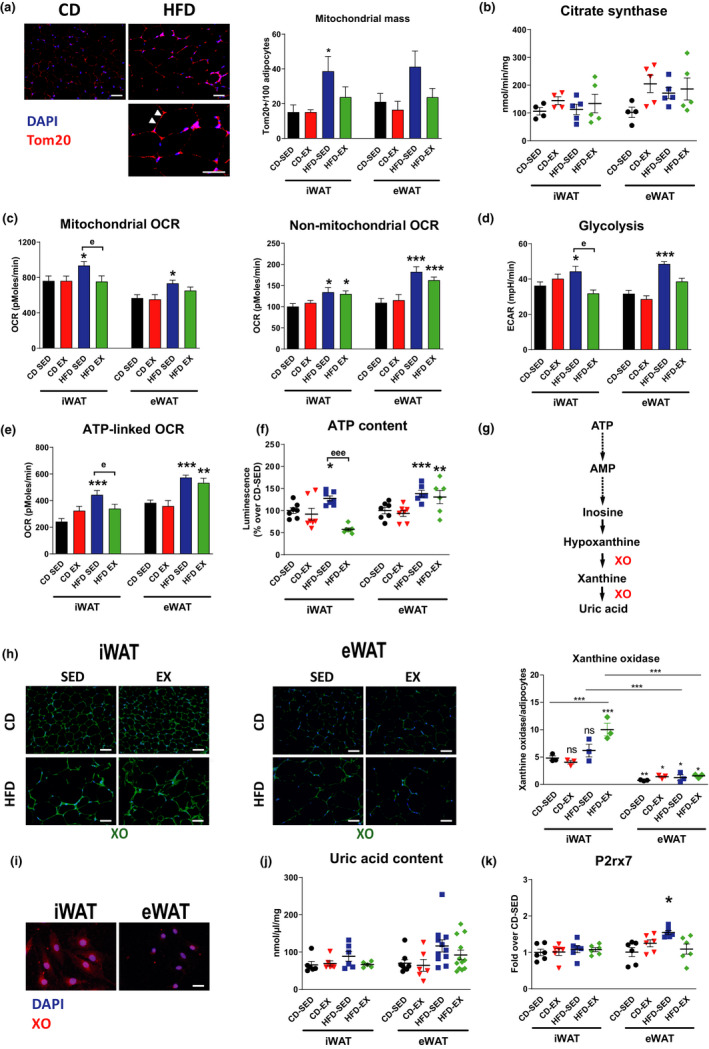
Adipose tissue senescence is associated with increased adipocyte bioenergetics. (a) Representative images of CD‐SED and HFD‐SED mice and quantification by immunofluorescence staining in iWAT and eWAT of translocase of outer mitochondrial membrane 20 (Tom20) signal in adipocytes (arbitrary unit, AU); *n* = 3–7 mice/group, magnification ×200, scale bar = 50 μm. Magnified view of positive cells (400×; bottom panel), scale bar = 50 µm. (b) Citrate synthase activity in iWAT and eWAT; *n* = 4–5 mice/group. (c) Mitochondrial and non‐mitochondrial oxygen consumption (OCR) determined by SeaHorse analysis in iWAT‐ and eWAT‐derived preadipocytes isolated from CD and HFD mice, sedentary (SED) or exercise (EX) groups; *n* = 4 independent experiments with five technical repeats. (d) Changes in extracellular acidification rate (ECAR), an index of glycolysis. Glycolysis is determined through measurements of the surrounding media before injection of compounds; *n* = 4 independent experiments with five technical repeats. (e) Changes in ATP‐linked oxygen consumption; *n* = 4 independent experiments with five technical repeats. (f) Intracellular ATP content in iWAT‐ and eWAT‐derived preadipocytes; *n* = 6–8 mice/group. (g) Flowchart of purine degradation. (h) Representative images and quantification of xanthine oxidase immunofluorescence (XO, green) in iWAT and eWAT. XO levels are expressed as the mean positive signal in percentage of adipocytes; *n* = 3 mice/group, magnification ×200, scale bar = 50 μm. (i) Representative images of XO immunofluorescence in iWAT‐ and eWAT‐derived preadipocytes; *n* = 3/group; magnification ×400, scale bar = 25 μm. (j) Uric acid concentration in iWAT and eWAT tissue lysates, *n* = 6–12 mice/group. (k) Gene expression analysis in iWAT and eWAT for purinergic receptor P2X, ligand‐gated ion channel, 7 (P2rx7). Results are expressed as fold mRNA change relative to CD‐SED values set to 1; *n* = 6 mice/group. Data are presented as original images (a, h–i), individual values plus mean (b, f, h, j–k), or mean ± SEM (a, c–e). Statistical significance was evaluated by one‐way ANOVA followed by Bonferroni correction. *p < 0.05; **p < 0.01; ***p < 0.001 for differences due to diet regimen within sedentary and exercise groups (* = diet effect) and ^e^ *p* < 0.05; ^ee^ *p* < 0.01; ^eee^ *p* < 0.001 for differences between sedentary and exercise groups fed the same diet (^e^ = exercise effect); ns: nonsignificant

Next, we assessed whether a 10‐week HFD intervention compromised mitochondrial homeostasis and whether exercise exerted beneficial effects on WAT bioenergetics (Stanford et al., 2015). Transcripts encoding for mitochondrial biogenesis and oxidative metabolism were downregulated by HFD in WAT (Figure S3A‐B). Specifically, the expression of the master regulators of mitochondrial biogenesis, peroxisome proliferator‐activated receptor gamma coactivator 1‐alpha (Pgc1α) and its downstream genes were downregulated by HFD in both WAT depots and preadipocytes (Figure S3A). HFD downregulated genes related to fatty acid oxidation, such as peroxisome proliferator‐activated receptor alpha (Pparα) and related genes in both WAT depots and preadipocytes (Figure S3B). Surprisingly, HFD increased iWAT and eWAT mitochondrial content of adipocytes as assessed by immunofluorescence of the mitochondrion‐specific marker Tom20 (Figure 3a) with unaltered citrate synthase activity in whole WAT (not shown) and in mature adipocytes (Figure 3b). Exercise normalized mitochondrial content, depressed genes related to mitochondrial biogenesis and WAT browning (Pgc1α, Prdm16, and Ucp1) in iWAT (Figure 3a; Figure S3A‐B). We further assessed mitochondrial expression profile in eWAT and found a HFD‐induced downregulation of several genes related to mitochondrial homeostasis, beta‐oxidation, morphogenesis (fission/fusion, autophagy, and apoptosis), as illustrated by a heatmap representation of their expression profiles (Figure S3C). These changes were associated with the downregulation of mitofusin2 (Mfn2) in both WAT (Figure S3D).

Next, we analyzed mitochondrial oxygen consumption (OCR) and glycolytic activity (extracellular acidification rate, ECAR) in real time by the Seahorse technology in preadipocytes (Figure S3E‐F). HFD increased mitochondrial and non‐mitochondrial OCR in preadipocytes derived from both WAT depots (Figure 3c). In addition, HFD enhanced glycolysis (ECAR) in preadipocytes of both WAT depots, while exercise restored it only in those derived from iWAT (Figure 3d).

Interestingly, ATP‐linked oxygen consumption was increased by HFD in preadipocytes from both iWAT and eWAT, suggesting a higher respiratory chain and ATP synthase activity (Figure 3e). In further support of increased energy production, we found higher ATP content in preadipocytes derived from both WAT depots of HFD sedentary mice, while exercise reduced ATP content in iWAT but not eWAT (Figure 3f).

This observation drew our attention to potential differences in the purine handling between these two fat depots. On the assumption that these fat pads have differential ability to catabolize accumulating high‐energy purines, we explored the first and only unidirectional purine catabolic enzyme, xanthine oxidase (XO), which catalyzes the oxidation of hypoxanthine to xanthine, and of xanthine to uric acid (Figure 3g). Interestingly, XO protein levels were considerably higher in iWAT than in eWAT per adipocyte, with further increase in iWAT adipocytes of HFD‐EX mice compared to that of CD mice (Figure 3h). Inherently higher XO expression in iWAT versus eWAT was confirmed at the stage of preadipocytes (Figure 3i). As a systemic consequence, plasmatic uric acid, the end‐degradation product of ATP, increased with HFD, but was normalized by exercise (Figure S3G). However, HFD did not increase uric acid content in either WAT depot (Figure 3j). HFD also increased the expression of the purinergic receptor P2X, ligand‐gated ion channel, 7 (P2rx7), a modulator of cell damage and ATP release, in eWAT but not in iWAT (Figure 3K).

**FIGURE 4:**
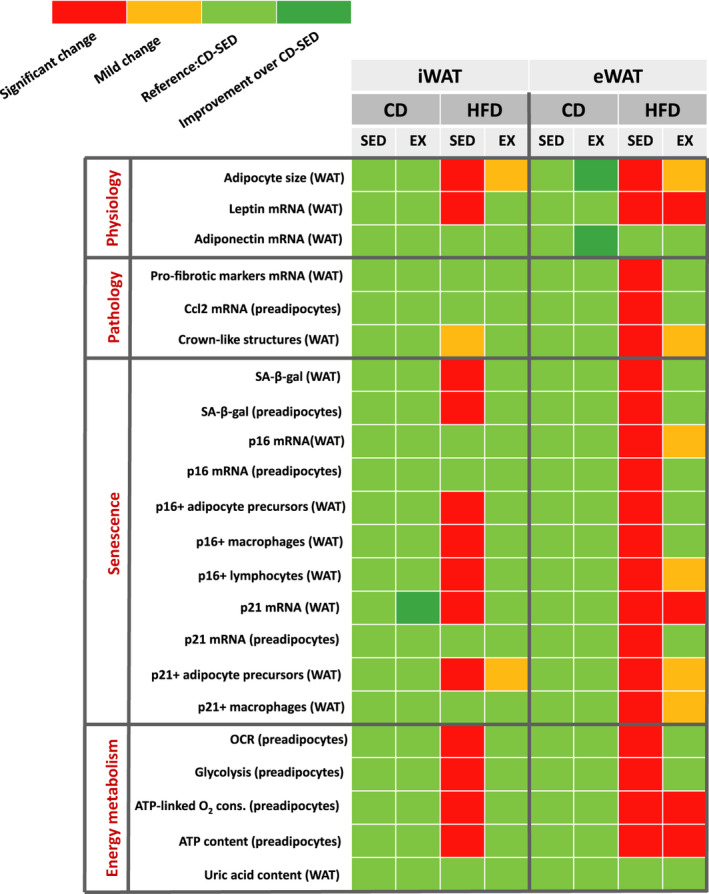
A summary table. The table displays the differences between iWAT and eWAT together with the effect of regimen and exercise in terms of adipose tissue function and dysfunction, senescence, and energy metabolism, including ATP and uric acid contents. Color code is determined by statistical significance

These results, highlighting the increase in HFD‐related ATP content in both WAT depots and the normalization by exercise only in iWAT, are summarized in Figure 4 together with the impact on WAT senescence.

### 2.4 Role of ATP in WAT senescence

**FIGURE 5:**
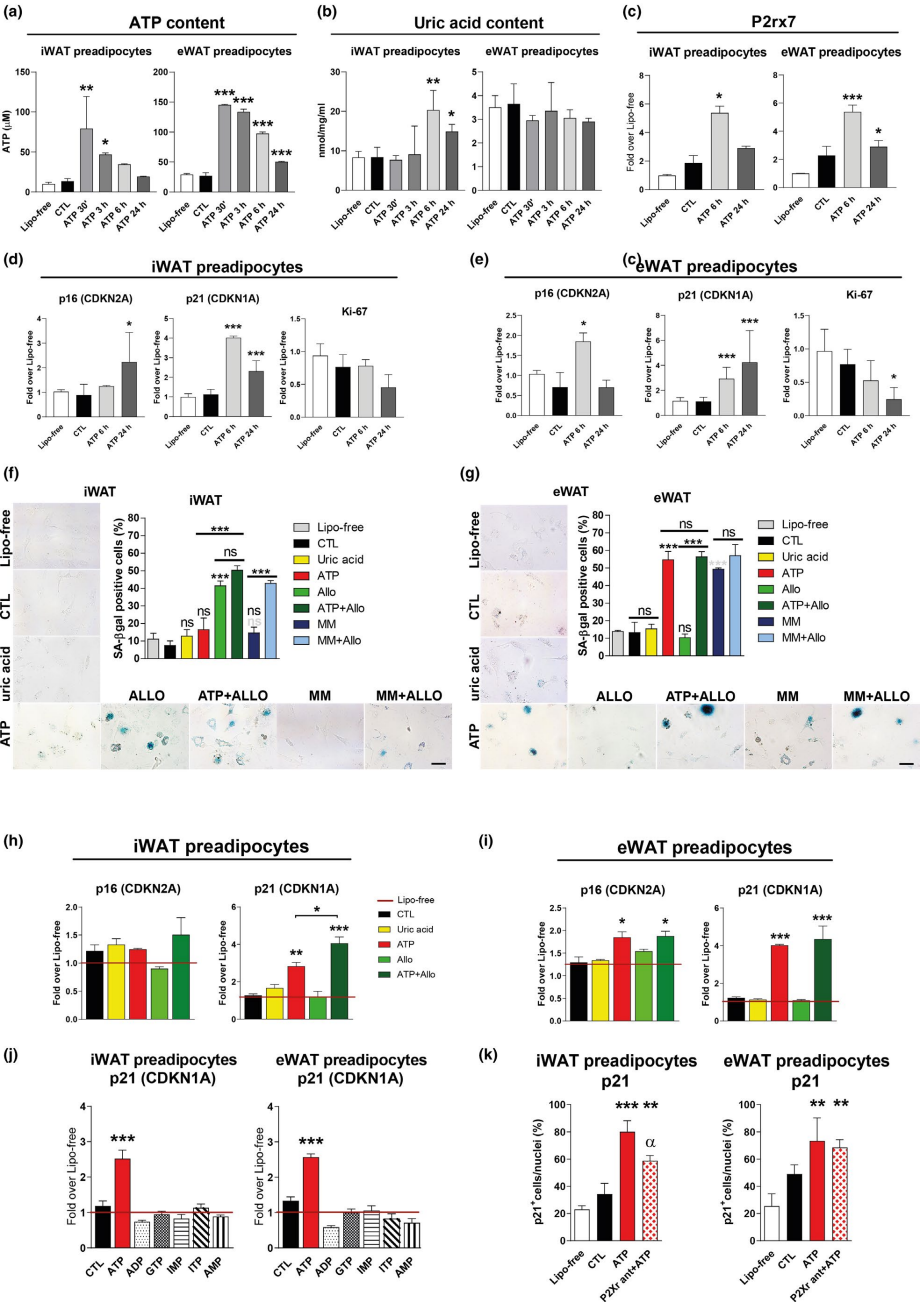
Role of ATP in adipose tissue senescence *in vitro*. (a–b) Intracellular ATP (a) and uric acid (b) levels in preadipocytes derived from iWAT and eWAT of CD‐fed mice treated with adenosine triphosphate (ATP)‐loaded liposomes (200 μM; for 30′–3 h–6 h–24 h) compared to two control groups: liposome‐free (lipo‐free) and liposome‐alone (CTL); *n* = 3–6/group. (c) Gene expression analysis in iWAT‐ and eWAT‐derived preadipocytes for purinergic receptor P2X, ligand‐gated ion channel, 7 (P2rx7) at 6 h and 24 h post ATP treatment. Results are expressed as fold mRNA change relative to Lipo‐free values set to 1; *n* = 3–4/group. (d–e) Gene expression analysis for senescence markers, p16 and p21, and proliferation marker, Ki‐67, in CD iWAT‐ and eWAT‐derived preadipocytes 6 h or 24 h post‐ATP treatment compared to liposome‐free (lipo‐free, red line) and liposome‐alone (CTL); *n* = 4–6/group. (f–g) Representative images of SA–β‐gal in CD iWAT‐ and eWAT‐derived preadipocytes stimulated for 6 h with uric acid, ATP ± allopurinol, high glucose/palmitate (metabolic media: MM) or MM in combination with allopurinol; magnification ×400, scale bar = 25 μm; *n* = 4–5/treatment. (h–i) Gene expression analysis for senescence markers, p16 and p21, in CD iWAT‐ and eWAT‐derived preadipocytes stimulated for 6 h with uric acid‐ or ATP‐loaded liposomes alone or in combination with allopurinol. For gene expression, results are expressed as fold mRNA change relative to Lipo‐free values set to 1; *n* = 3/group. (j) Gene expression analysis for p21 in CD iWAT‐ and eWAT‐derived preadipocytes 24 h after treatment with ATP‐, ADP‐, GTP‐, IMP‐, ITP‐, and AMP‐loaded liposomes compared to liposome‐free (lipo‐free, red line) and liposome‐alone (CTL); *n* = 3/group. (k) Quantification of immunofluorescence staining for p21 (red), counterstained with DAPI (blue), in CD iWAT‐ and eWAT‐derived preadipocytes stimulated with ATP‐loaded liposomes for 24 h, after pre‐treatment with P2X r antagonist, compared to liposome free (lipo‐free) and liposome alone (CTL). Results are expressed as nuclear p21 in percentage of total nuclei (*n* = 3/group). Data are presented either as original images (F‐G) or mean ± SEM (a–k). Statistical significance was evaluated by one‐way ANOVA followed by Bonferroni correction. *p < 0.05; **p < 0.01; ***p < 0.001 compared to liposome alone (CTL) or as indicated; ns: nonsignificant; gray symbols above columns: significance vs. Lipo‐free. ^α^ *p* < 0.05 for the effect of P2X r antagonist between ATP‐treated cells

Since we identified a positive correlation between the early increase in ATP content and WAT senescence (Figure 1h), we investigated the specific role of intracellular ATP in driving WAT senescence. Hence, we assessed SA‐β‐gal activity, p16, p21, and Ki‐67 expression in iWAT‐ and eWAT‐derived primary preadipocytes treated with ATP‐loaded liposomes. First, ATP peaked at 30 min after treatment in both iWAT and eWAT preadipocytes. In iWAT but not eWAT, ATP content rapidly declined with a concomitant increase in uric acid (Figure 5a–b). This is consistent with the difference in XO protein expression in the respective WAT depots (Figure 3h–i), while purinergic receptor expression increased in both iWAT‐ and eWAT preadipocytes in a similar manner at 6 h of ATP stimulation (Figure 5c). ATP‐loaded liposomes induced p21 expression at 6 h after treatment in both iWAT and eWAT, and p16 expression at 6 h in eWAT and delayed at 24 h in iWAT (Figure 5d–e). Ki‐67 expression significantly decreased at 24 h only in eWAT, in parallel with the induction of SASP factors (Figure 5d–e; Figure S5A). Thus, we further evaluated ATP‐inducible senescence compared to either liposome free, liposome control, or uric acid‐loaded liposomes after 6‐h treatment (Figure 5f–i). Senescence occurred only after delivery of ATP, but not uric acid, in comparison with liposome‐free and liposome‐alone control conditions. Of note, ATP‐induced senescence was more pronounced in eWAT than in iWAT (Figure 5f–i). To prove the role of ATP accumulation in initiating WAT senescence in the context of high energy state, we prevented the degradation of ATP with allopurinol, a XO inhibitor, in both ATP‐ and in high glucose/palmitate‐ (metabolic media: MM, to mimic caloric overload *in vitro*) treated preadipocytes (Figure 5f–g). Notably, allopurinol aggravated senescence in ATP‐ and MM‐treated preadipocytes derived from iWAT but not eWAT, where we found low levels of XO expression. Specifically, MM increased SA‐β‐gal positivity in eWAT, but not in iWAT preadipocytes, in line with the differences in XO levels between the two fat depots (Figure 3h). Allopurinol increased p21 expression in ATP‐treated preadipocytes derived from iWAT alone without further increase in those of eWAT, while p16 only increased in eWAT‐derived preadipocytes without impact by allopurinol (Figure 5h–i). Similar senescence features were obtained in treating 3T3‐L1 adipocytes with ATP‐ and uric acid‐loaded liposomes, lending support to the critical role of ATP, but not uric acid, in inducing WAT senescence (Figure S5b–c). To strengthen the specific role of ATP among purines to induce WAT senescence, we treated iWAT and eWAT‐derived preadipocytes with a variety of other purines (ADP, GTP, IMP, ITP, and AMP), which, as opposed to ATP, all failed to induce senescence as assessed by p21 expression (Figure 5j). To exclude a major role for extracellular ATP in inducing senescence, we treated preadipocytes with ATP‐loaded liposomes while specifically blocking the purinergic receptor P2X and observed only a minor albeit significant decrease in p21 expression in iWAT, while no such changes in eWAT (Figure 5k).

**FIGURE 6:**
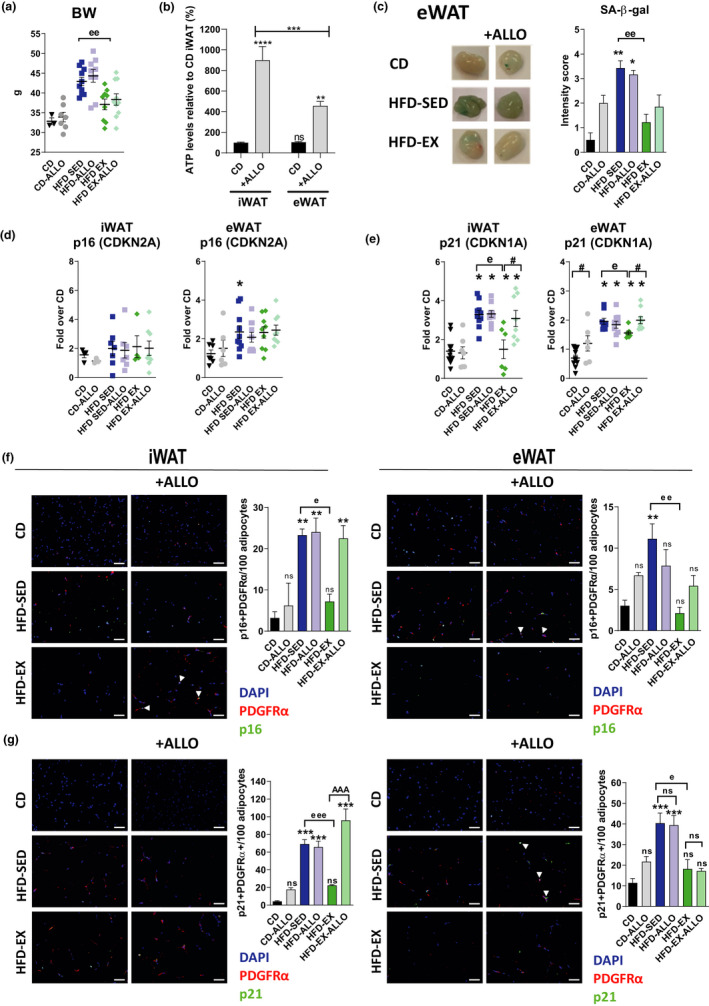
Role of ATP in adipose tissue senescence *in vivo*. (a) Body weight (BW) of six groups of mice: control diet (CD), CD treated with allopurinol (CD‐ALLO), HFD‐sedentary (HFD‐SED), HFD‐sedentary treated with allopurinol (HFD‐ALLO), HFD‐exercise (HFD‐EX) and HFD‐exercise treated with allopurinol (HFD EX‐ALLO); *n* = 5–10 mice/group. (b) Relative ATP levels in preadipocytes derived from iWAT and eWAT of CD and CD‐ALLO mice, *n* = 4–6 mice/group. (c) Representative images and quantification of SA–β‐gal in eWAT in mice as described under A; *n* = 5 mice/group. (d–e) Quantitative RT‐PCR analysis of p16 (d) and p21 (e) in iWAT and eWAT of mice described under A; *n* = 5–10 mice/group. (f–g) Representative images and quantification by immunofluorescence staining in iWAT (f) and eWAT (g) of co‐localization with p21 (green) and PDGFRα (red). Sections were counterstained with DAPI (blue). Double positive cells are expressed as the mean number of positive cells in percentage of adipocytes; *n* = 4–5 mice/group, magnification ×200, scale bar = 50 μm. Data are presented as original images (c, f–g), individual values plus mean (a, d–e) or mean ± SEM (b–c, f–g). Statistical significance was evaluated by one‐way ANOVA followed by Bonferroni correction. *p < 0.05; **p < 0.01 ***p < 0.001 above columns indicate comparison to CD (* = diet effect) or comparison is indicated; ^e^ *p* < 0.05; ^ee^ *p* < 0.01 for differences between sedentary and exercise groups fed the same diet (^e^ = exercise effect); ^#^ *p* < 0.05 for the effect of allopurinol treatment between groups; ns: nonsignificant

Since these *in vitro* data strongly suggested a causative role for intracellular ATP in the induction of WAT senescence, we inhibited the degradation of ATP by allopurinol administration after 5‐week HFD *in vivo*, alone or in combination with exercise, and compared readouts with CD (Figure S6A). Allopurinol, as opposed to exercise, did not affect body weight, insulin sensitivity (ITT), and glucose tolerance (GTT) in HFD group (Figure 6a and Figure S6B–C). Importantly, we found that *in vivo* allopurinol treatment increased intracellular ATP content in preadipocytes isolated from either iWAT or eWAT, which was of greater magnitude in iWAT (Figure 6b). An increased reliance on ATP disposal in iWAT compared to eWAT is further suggested by delayed elevation of ATP levels in preadipocytes derived from iWAT after *in vivo* HFD feeding (Figure S6D). Consistent with low XO expression in eWAT (Figure 3h) allopurinol did not further increase the SA‐β‐gal activity in eWAT of HFD mice (Figure 6c). Consistent with our exercise cohort (Figure S2), HFD induced p16 gene expression only in eWAT with no further change with allopurinol (Figure 6d). By contrast, p21 mRNA levels were increased by HFD with or without allopurinol in both fat pads and rescued by exercise. This rescue was blocked only in iWAT with allopurinol (Figure 6e). With regard to senescence in PDGFRα+ adipocyte precursors, HFD +/‐ allopurinol increased the frequency of p16+ and p21+ adipocyte precursors in both iWAT and eWAT rescued by exercise. Notably, allopurinol prevented this rescue by exercise in iWAT alone (Figure 6f–g; Figure S6 *Fbis*; Figure S6 *Gbis*). The effectiveness of allopurinol treatment was confirmed by a 15% decrease in uric acid plasma levels (Figure S6E).

Altogether, these results strongly indicate a specific role for ATP but neither other purines including uric acid, in the induction of WAT senescence in a 10‐week HFD intervention. Remarkably, addition of allopurinol reduced ATP degradation, thus amplifying the prosenescent effect of ATP in caloric overload *in vitro* and *in vivo* in iWAT with high XO expression and mimicked HFD‐like, prosenescent effects in CD mice.

## 3 DISCUSSION

Our data highlight the accumulation of senescent cells within both visceral and subcutaneous WAT as a very early event occurring two weeks after HFD initiation in parallel with metabolic alterations independent of systemic inflammation or WAT fibrosis. Furthermore, we identify HFD‐induced ATP overproduction by increased glycolysis and oxidative phosphorylation, as a mechanism initiating WAT senescence. Exercise prevents metabolic alterations independent of major body weight and adiposity reduction and ameliorates senescence mainly in iWAT. This finding is consistent with the high expression of xanthine oxidase in iWAT, which allows for ATP degradation and explains the full rescue of senescence during exercise in this fat depot.

### 3.1 WAT senescence occurs early after HFD initiation

In the context of caloric overload, the energy storage in WAT reaches a threshold that initiates a cascade of events ultimately leading to chronic low‐grade inflammation, increased generation of ROS and pathological consequences, including insulin resistance and remote organ damage (Minamino et al., 2009; Palmer et al., 2019; Ternacle et al., 2017). While most of the literature evaluates long‐term HFD, we focused our investigation on 10‐week HFD in adult mice. Although we neither observed WAT fibrosis, nor local or systemic inflammation, HFD‐induced glucose intolerance, insulin resistance, and leptin production consistent with increased fat mass and adipocyte hypertrophy together with organization of immune cells in crown‐like structures (Murano et al., 2008).

From a detailed kinetic during a 10‐week HFD exposure, our main observation is that cellular senescence represents one of the earliest events occurring in WAT, starting two weeks after the initiation of HFD as demonstrated by the increase of a combination of senescence markers (SA‐β‐gal activity, CDKN1a, and CDKN2a) (Ogrodnik et al., 2019) together with adipocyte hypertrophy, as recently reported in other cell types (Anderson et al., 2019; Biran et al., 2017; Neurohr et al., 2019).

Moreover, we show that HFD impacted on various cell types of adipose tissue. Specifically, senescence occurred in different WAT cell populations, including mature adipocytes, preadipocytes, adipocyte progenitors, and immune cells. Interestingly, the senescence phenotype was maintained in differentiated HFD‐derived preadipocytes, reflecting the *in vivo* characteristics of parental tissue (Hausman et al., 2008). This finding is important considering the impact of WAT senescence on systemic disorders and lifespan (Baker et al., 2016).

Of note, we did not observe any tissue induction of classical pro‐inflammatory SASP factors (CCL2, TNFa, IL6, IL10, and PAI1) (Coppe et al., 2008; Munoz‐Espin & Serrano, 2014; Saker et al., 2016) as opposed to longer (i.e., 30 vs. 10 weeks in our model) HFD exposure in older (12–13 vs. 7 months) animals (Schafer et al., 2016). This is consistent with a recent report on the dynamic changes of SASP at varying intervals after *in vitro* senescence induction in other cell types (Hernandez‐Segura et al., 2017). Instead, we observed an upregulation of several SASP‐associated chemokines (Mcp1) and profibrotic cytokines (Tgfb1, Fn1, and Timp1) in eWAT but not iWAT. Indeed, MCP‐1 is secreted by senescent cells and has recently been correlated with biological age in mammals (Yousefzadeh et al., 2018), while TGFβ promotes matrix remodeling in remote organs (Sawaki et al., 2018). Our current data and recent observations (Sawaki et al., 2018; Ternacle et al., 2017) support the premise that senescent WAT is an important source of profibrotic cytokines, known to alter tissue homeostasis prior to the induction of a pro‐inflammatory secretome. This profibrotic secretome contributes to organ remodeling, physical dysfunction, and premature aging (van Deursen, 2014; Munoz‐Espin & Serrano, 2014; Sawaki et al., 2018).

### 3.2 Increased ATP content initiates WAT senescence without mitochondria dysfunction

Mitochondria are important for senescence and the development of SASP (Correia‐Melo et al., 2016; Passos et al., 2010). In the context of high calorie intake, mitochondria increase ROS generation as a toxic by‐product of oxidative phosphorylation. Our data are consistent with this observation since our 10‐week HFD regimen resulted in marginally increased mitochondrial ROS production in WAT exerting a potential trophic effect (Lee et al., 2009). Additionally, mitochondria play a critical role in maintaining cellular activities by generating energy in the form of ATP. In HFD sedentary mice, our data show an elevated ATP content in both iWAT and eWAT preadipocytes, but exercise normalized it only in iWAT. Thus, we explored the possibility of increased purine catabolism in iWAT as a potential means to alleviate the burden of abnormally high energy content. First, we observed substantially higher expression of xanthine oxidase, the enzyme controlling purine degradation, in iWAT compared to that in eWAT. Moreover, XO uses molecular O_2_ as an essential cofactor, which is provided by exercise hyperemia known to improve blood flow in the neighboring iWAT. The combination of high XO expression, HFD increased levels of substrate (i.e., ATP) for purine degradation and O_2_ cofactor is consistent with the difference between iWAT and eWAT senescence prevention by exercise. Furthermore, we confirm the noxious impact of elevated ATP levels by blocking its degradation using allopurinol, which antagonized beneficial effects of exercise in iWAT of HFD mice and induced WAT senescence in CD mice. Finally, we identify the specific effect of ATP among other purines (ADP, AMP, GTP, IMP, and ITP) since their administration failed to induce WAT senescence. We also show an HFD‐induced expression of P2rx7, a modulator of cell senescence (Cho et al., 2014) increased in obesity, restricted to eWAT.

Since high concentrations of ATP and its end‐degradation products have been shown to be endogenous “danger‐associated molecular patterns,” DAMPs (Basisty et al., 2020; Nakahira et al., 2015), we further demonstrated that ATP treatment‐induced senescence in both primary preadipocyte and 3T3‐L1 adipocyte culture, while uric acid did not. Indeed ATP, but not uric acid‐loaded liposomes, rapidly induced an increase in SA‐β‐gal activity and an upregulation of p16 and p21, which was altogether more pronounced in eWAT‐derived preadipocytes. Tight control of ATP is also beneficial in obesity‐related metabolic alterations as demonstrated in a recent report on the role of cardiolipin to regulate ATP production (Prola et al., 2021). The role of increased ATP in driving cellular senescence is considered specific to adipose tissue and early stages of HFD when mitochondrial integrity is uncompromised. Since low levels of ATP have been linked to senescence induction in human fibroblasts (Wang et al., 2003; Zwerschke et al., 2003), human endothelial cells (Unterluggauer et al., 2008), and in replicative senescence models (Hanzelmann et al., 2015), it will be interesting to explore where ATP‐induced senescence stands in the heterogeneous spectrum of senescent phenotypes (Casella et al., 2019; Hernandez‐Segura et al., 2017, 2018; Ogrodnik et al., 2019) and how it may be exploited for therapeutic benefit.

### 3.3 Exercise exerts a “senostatic” action preventing WAT senescence during HFD

Our study extends an important observation that exercise prevents adipose tissue senescence in HFD (Schafer et al., 2016) and adds mechanistic clues to a triggering role for ATP. The significance of our present findings is further highlighted by the lack of confounding factors known to interfere with senescence, such as physiological age (Sawaki et al., 2018) and weight loss (Ma et al., 2020), in our study design.

Exercise promotes mitochondrial biogenesis and improves function in multiple organs including the heart (Judge & Leeuwenburgh, 2007). In the current experimental model of HFD, however, we show that exercise reduced global WAT respiration, mitochondrial ATP‐linked oxygen consumption, and glycolysis, ultimately resulting in reduced ATP accumulation in iWAT but not in eWAT. Moreover, the higher expression of XO, the rate‐limiting enzyme in purine catabolism, in iWAT may facilitate depletion of ATP upon exercise in HFD mice. It remains to be explored whether improved insulin sensitivity in obesity by exercise is specifically mediated by alleviation of excessive energy generation. These findings are in line with the normalization of HFD‐induced increase in mitochondrial mass, a pattern closely reflecting observations in other models of senescence (Korolchuk et al., 2017; Stab et al., 2016). As ATP acts as a danger signal on immune cells (Faas et al., 2017), our data show that exercise‐dependent decrease in ATP levels reduced p16 and p21 expression in WAT macrophages, which may improve the clearance of senescent cells. Our data are in line with a recent observation showing that restraining mitochondrial ATP synthesis by a moderate respiratory chain complex IV inhibitor might alleviate age‐associated disorders (Tavallaie et al., 2020).

### 3.4 Clinical impact

Given the worldwide obesity epidemics, there is an increasing interest in public health programs encouraging healthy eating behavior and physical activity. Aerobic training is known to have a beneficial impact at different life stages on several aspects of human health, such as cognitive function, cardiovascular, endocrine, and immune responses. Importantly, the benefit of exercise has been demonstrated in pathological conditions, including diabetes, cancer, and cardiovascular disorders (Myers, 2003). Here, we add mechanistic insights to the role of exercise in preventing both cellular senescence and metabolic alteration in the context of obesity, reinforcing the concept that adipose tissue dysfunction affects glucose homeostasis (Pini et al., 2016). Indeed, we demonstrate that exercise improves WAT cellular senescence, adipokine dysregulation, and adipocyte bioenergetics independent of weight loss, favoring the recovery of glucose homeostasis in obesity. Therefore, exercise through the control of local ATP seems an interesting approach to prevent the induction of cellular senescence and its potentially deleterious consequences. In summary, exercise is an excellent senostatic strategy without any side effect, for preventing obesity‐associated senescence in WAT.

## 4 EXPERIMENTAL PROCEDURES

### 4.1 Animals

Five‐month‐old C57/BL6JRj mice (WT, Janvier Labs, France) were subjected to chow diet (CD) or HFD (60% calories from fat, D12492, irradiated; Research Diets Inc., New Brunswick, NJ) for 1, 2, 5, and 10 weeks to assess the kinetics of epididymal white adipose tissue (WAT) senescence.

In a separate set of experiments, CD and HFD mice were followed up to 10 weeks. After 5 weeks on the respective diets, mice were randomly assigned to CD‐sedentary (CD‐SED), CD‐exercise (CD‐EX), or HFD‐sedentary (HFD‐SED), HFD‐exercise (HFD‐EX); *n* = 15/group (Figure S1E). The exercise consisted of two swimming sessions/day for 5 days/week during 4 weeks (Derumeaux et al., 2008); see Appendix S1 for details. Mice were 7‐month‐old at necropsy. To avoid any bias related to differences in body weight, the whole investigation was conducted in mice with closely matched body weights and adiposity levels, as measured at the end of the protocol (*n* = 8/group; Table S1).

In a separate set of experiments, 3‐month‐old p16^LUC^ heterozygote mice (obtained from N E Sharpless (Chapel Hill, NC, USA) and bred at CNRS‐TAAM, France), harboring a knock‐in of the luciferase gene into the Cdkn2a locus (Sorrentino et al., 2014), were exposed to CD and HFD for 10 weeks. This model aimed to explore *in vivo* the HFD‐induced transcription of p16INK4a, a marker of senescence.

In a separate set of experiments, CD and HFD mice (5‐month‐old, *n* = 10) were treated with allopurinol (1mM in the drinking water) (Bravard et al., 2011), starting 5 weeks after the initiation of HFD, alone or in combination with exercise, up to 10 weeks of respective dietary regimes. They were compared with a control group of CD male mice. (Figure S6A).

All experiments were performed in accordance with the guidelines of our Institutional Animal Care and Use Committee (IACUC). The protocol was approved by the IACUC of the French National Institute of Health and Medical Research, U955, Creteil, France (ComEth 15–001).

### 4.2 In vivo procedures

During follow‐up animals underwent weekly body weight (BW) measurements. Daily food consumption was monitored over 1 week in all groups. Insulin (ITT) and glucose (GTT) tolerance tests were performed at baseline, before exercise and at the end of exercise intervention as described previously (Sawaki et al., 2018).

### 4.3 Plasma and tissue collection and measurements

On the day of the sacrifice, mice were not fasted and CD and HFD exercise groups had the last swimming session 24 h before. Blood was collected and processed as described (Sawaki et al., 2018). Subcutaneous (inguinal, iWAT), visceral (epididymal, eWAT), and perirenal (pWAT) WAT samples were collected, weighed, and immediately processed for further analysis. Plasma leptin and adiponectin were quantified by ELISA (R&D Systems, Minneapolis, MN, USA). Conditioned media (eWAT secretome) and plasma were analyzed for a selected panel of pro‐inflammatory mediators (Campisi, 2005; Coppe et al., 2008; Munoz‐Espin & Serrano, 2014; Saker et al., 2016), using a Luminex Bio‐Plex cytokine assay kit (Bio‐Rad, USA). Plasma and WAT uric acid was quantified by a colorimetric assay kit according to the manufacturer's instructions (BioVision, Milpitas, CA, USA). Adiposity index was calculated by the ratio between the sum of three WAT weights and body weight.

### 4.4 Histological assessment

Sections of iWAT and eWAT were stained with hematoxylin & eosin for adipocyte size measurements. Sirius Red and immunofluorescent staining procedures were performed as reported previously (Sawaki et al., 2018). Antibodies are listed in Table S2. The number of biologically distinct samples and replicates, which were averaged for each mouse, is reported in Table S3.

### 4.5 Primary cell culture

Preadipocytes were isolated from iWAT and eWAT stroma vascular fraction (SVF) and differentiated in an adipogenic culture media after two passages to eliminate non‐preadipocyte cell contamination. In detail, WAT was aseptically minced and incubated with collagenase Type II (1 mg/mL, Sigma) at 37℃ for 45 min. After digestion, serum‐containing medium was added to the suspension and filtered through a 100 μm cell strainer. SVF cells were centrifuged for 10 min at 350 g and suspended in pre‐warmed adipogenic culture media, consisting of DMEM high glucose with Glutamax (Gibco) supplemented with 10% newborn calf serum (Thermo fisher), 2.4 nM human insulin (Sigma), and 1% antibiotic solution (Pen‐Strep solution, Sigma). To induce differentiation, SVF cells were seeded at near‐confluence and medium was replenished every day to allow selection by adhesion during the first 3 days and then changed every 2 days. SVF cells were cultured in an atmosphere of 5% CO_2_ and 20% O_2_ at 37℃. Based on preliminary experiments, day 7 was the time of differentiation used for all experiments.

### 4.6 SA‐β‐galactosidase activity

For senescence assays, portions of iWAT and eWAT depots freshly dissected or differentiated preadipocytes derived from these animals were fixed with 1% PFA, and then incubated with SA‐β‐Gal staining solution (1 mg/ml Ultrapure X‐gal, Sigma‐Aldrich, France) (Itahana et al., 2007; Sawaki et al., 2018) at 37℃. For β‐galactosidase activity in tissue, a score of relative intensity of the signal (0 = absent, 1 = low, 2 = medium, 3 = high, and 4 = very high) was used.

### 4.7 Primary cell metabolic rate measurements

For bioenergetic assessment, the SeaHorse Mitochondrial Stress Test was performed on iWAT‐ and eWAT‐differentiated adipocytes (20,000 cells/well) using the XF24 Extracellular Flux Analyzer (Agilent Technologies, Santa Clara, CA, USA). Oxygen consumption rate (OCR), indicative of mitochondrial activity, and extracellular acidification rate (ECAR), an index of glycolysis, were determined as described previously (Foresti et al., 2015).

### 4.8 ATP measurements

Differentiated primary preadipocytes and 3T3‐L1 were lysed and intracellular ATP content measured by the ATP‐lite assay kit (Perkin Elmer, Villebon‐sur‐Yvette, France) (Braud et al., 2018).

### 4.9 Cell culture treatments

Primary preadipocytes were isolated from indicated WAT depots of 5‐month‐old CD male mice. At differentiation, cells were incubated at different time points (for the time course: 30 min‐3 h‐6 h‐24 h or as specified in the figure legend) with adenosine triphosphate (ATP)‐ and uric acid‐loaded liposomes (200 μM, Sigma‐Aldrich, France) and were compared to two control conditions: liposome‐free (lipo‐free) and liposome‐alone (CTL). In a separate set of experiments, preadipocytes were incubated for 24 h with liposomes loaded with other purines (200 μM of ADP, AMP, GTP, IMP, and ITP—Sigma‐Aldrich, France) and compared with controls and ATP. To block the purinergic receptor P2X, preadipocytes were pretreated with an antagonist (A‐438079 hydrochloride hydrate, Sigma‐Aldrich, France) 3 h before ATP‐loaded liposome stimulation.

In addition, to assess the role of ATP as a trigger of senescence, we performed interference experiments by co‐administration of the xanthine oxidase (XO) inhibitor allopurinol. In this set of experiments, preadipocytes were subjected for 6 h to *in vitro* caloric overload in the form of either high glucose/palmitate (Metabolic media: MM) or ATP‐loaded liposomes and co‐administration of allopurinol (1 mM, xanthine oxidase inhibitor, ab142565, Abcam).

Fully differentiated 3T3‐L1 adipocytes, cultured in an atmosphere of 5% CO_2_ and 20% O_2_ at 37℃, were incubated for 24 h with ATP or uric acid (200 μM, Sigma‐Aldrich, France). ATP and the other molecules were encapsulated in liposomes (Sigma‐Aldrich, France) (Braud et al., 2018).

### 4.10 Superoxide measurement

Cytosolic and mitochondrial ROS levels were measured in differentiated preadipocytes (7000/well) by quantification of CellROX (10 µM) and MitoSOX (5 µM) fluorescence intensity levels, respectively. Fluorescence readings were performed using a microplate reader (Tecan, Männedorf, Switzerland).

### 4.11 Quantitative real‐time PCR

Total RNA was isolated from iWAT and eWAT, differentiated preadipocytes, and 3T3‐L1 adipocytes using RNeasy Lipid Tissue Mini Kit (Qiagen, Valencia, CA, USA). Quantification was performed with Qubit RNA HS Assay Kit (Life Technologies, Thermo Fisher Scientific, MA, USA). First‐strand DNA synthesis was realized using 0.25–0.5 ng total RNA and qPCR was performed and analyzed as described (Sawaki et al., 2018). Taqman assays are reported in Table S2.

### 4.12 RNA profiling

PAMM‐087ZA RT^2^ Profiler™ PCR Array (Qiagen, Valencia, CA, USA) was performed on 600 ng of total RNA from eWAT. Real‐time PCR was then performed using Brillant II SYBR Green QPCR Master Mix (Agilent) and Mx3005 Pro thermocycler (Agilent).

### 4.13 Statistical analyses

Statistical analyses were carried out using GraphPad Prism 5 (La Jolla, CA) and the R environment for statistical computing. Data are reported as either as individual values plus mean or mean ± SEM. Comparisons between multiple groups were done using one‐way analysis of variance (ANOVA) followed by Bonferroni correction. Time‐dependent evolution of readouts was analyzed by two‐way ANOVA. *p*‐values <0.05 were considered as significant. All images were processed with the ImageJ (NIH, https://imagej.nih.gov/ij/). To evaluate the importance of the changes affecting major biological annotations that characterize the WAT functional signature under the effect of HFD, we measured the iWAT and eWAT expression profile of a panel of 22 genes illustrating adipocyte function, inflammation, tissue remodeling, and senescence. A random forest supervised learning algorithm (“randomForest” R package, version 4.6‐12) was applied iteratively to evaluate and rank the relative discriminative power associated with changes in these gene expression profiles induced by HFD in iWAT and eWAT. To assess the relative discriminative power associated with each measured expression profile, we averaged the feature importance provided by the random forest algorithm over 10,000 successive iterations performed for each of the analyzed situations. The individual discriminative power of each transcriptional profile was expressed as percentage of the total discriminative power of the panel of selected transcriptional descriptors. The resulting values were plotted after grouping them by functional category and ranking the available categories in a decreasing order of their strongest transcriptional descriptor, to illustrate the contextual importance of the functional changes induced by diet in the two WAT depots. Separately, the expression of a panel of several mitochondria genes was quantified by using RT2 ProfilerTM PCR Array. A linear regression model was used to test for differential expression in relation to diet. Significant transcriptional changes were then annotated in relation to three main themes characterizing mitochondrial function (i.e., morphogenesis, homeostasis, and beta‐oxidation) and illustrated graphically using a heatmap representation.

## CONFLICT OF INTEREST

None declared.

## AUTHOR CONTRIBUTIONS

M.P., G.C., and D.S. helped design the study, performed experiments, collected and analyzed the data, implemented follow‐up experiments, and wrote the manuscript. Z.M., L.B., T.D., and R.M. performed specific experiments and collected data; G.M. provided expertise in relation to adipose tissue fibrosis. C.M., N.B., and A.B.S. provided expertise for Seahorse data interpretation and specific experiments on mitochondrial function, and revised the manuscript. R.M. and R.F. helped with Seahorse experimental design and analysis, and revised the manuscript. C.H. helped analyze and interpret data, performed specific statistical tests, and revised the manuscript. G.D. designed the study, interpreted data, wrote and reviewed the manuscript, and provided study resources. All authors approved the final version of manuscript.

## Supporting information

Appendix S1

## Data Availability

All data are stored in a centralized and secured Clinical and Translational Research data Repository (CARMMA CTRDR) available at IMRB. Data and resource sharing are available upon request and presentation of a protocol to RHU CARMMA consortium.
