# Supplementary material for: Adipose tissue senescence is mediated by increased ATP content after a short‐term high‐fat diet exposure
Source: Aging Cell. 2021 Jul 18;20(8):e13421. doi: 10.1111/acel.13421 (PMC8373332; doi:10.1111/acel.13421)
Supplement: Supplementary file 1 — Appendix S1 [file ACEL-20-e13421-s001.pdf]

Supportive information

Adipose tissue senescence is mediated by increased ATP content after a short-term high fat diet exposure

Maria Pini PhD[1,*], Gabor Czibik MD, PhD[1,*], Daigo Sawaki MD, PhD[1,*], Zaineb Mezdari MS[1], Laura Braud PhD[2], Thais Delmont BSC[1], Raquel Mercedes MD[1], Cécile Martel PhD[4], Nelly Buron PhD[4], Geneviève Marcelin PhD[5], Annie Borgne-Sanchez PhD[4], Roberta Foresti PhD[2], Roberto Motterlini PhD[2], Corneliu Henegar MD, PhD[1], Geneviève Derumeaux MD, PhD[1]

[1] INSERM U955, Université Paris-Est Créteil (UPEC), AP-HP, Department of Physiology, Henri Mondor Hospital, FHU SENEC 94100 Créteil, France
[2] INSERM U955, Université Paris-Est Créteil (UPEC), Faculty of Medicine, IMRB, F-94010 Créteil, France
[3] AP-HP, Department of Cardiology, Henri Mondor Hospital, FHU SENEC, Créteil, France
[4] Mitologics S.A.S., Université Paris-Est Créteil (UPEC), Créteil, France
[5] Sorbonne Universities, INSERM UMR_S 1269, Nutriomics, F-75013, Paris, France

*Authors equally contributed to the work

Address for correspondence:

Geneviève Derumeaux, INSERM U955 Université Paris-Est Créteil (UPEC), 51 Av de Lattre de Tassigny 94100 Créteil, France, Tel: +33 1 49 81 Fax: +33 1 49 81 26 67 E-mail: genevieve.derumeaux@inserm.fr

Keywords: obesity, exercise, adipose tissue senescence, bioenergetics, ATP, xanthine oxidase

Supportive information

- **Supplementary figures**
- **Supplementary methods**
- **Supplementary tables: 1, 2, 3**

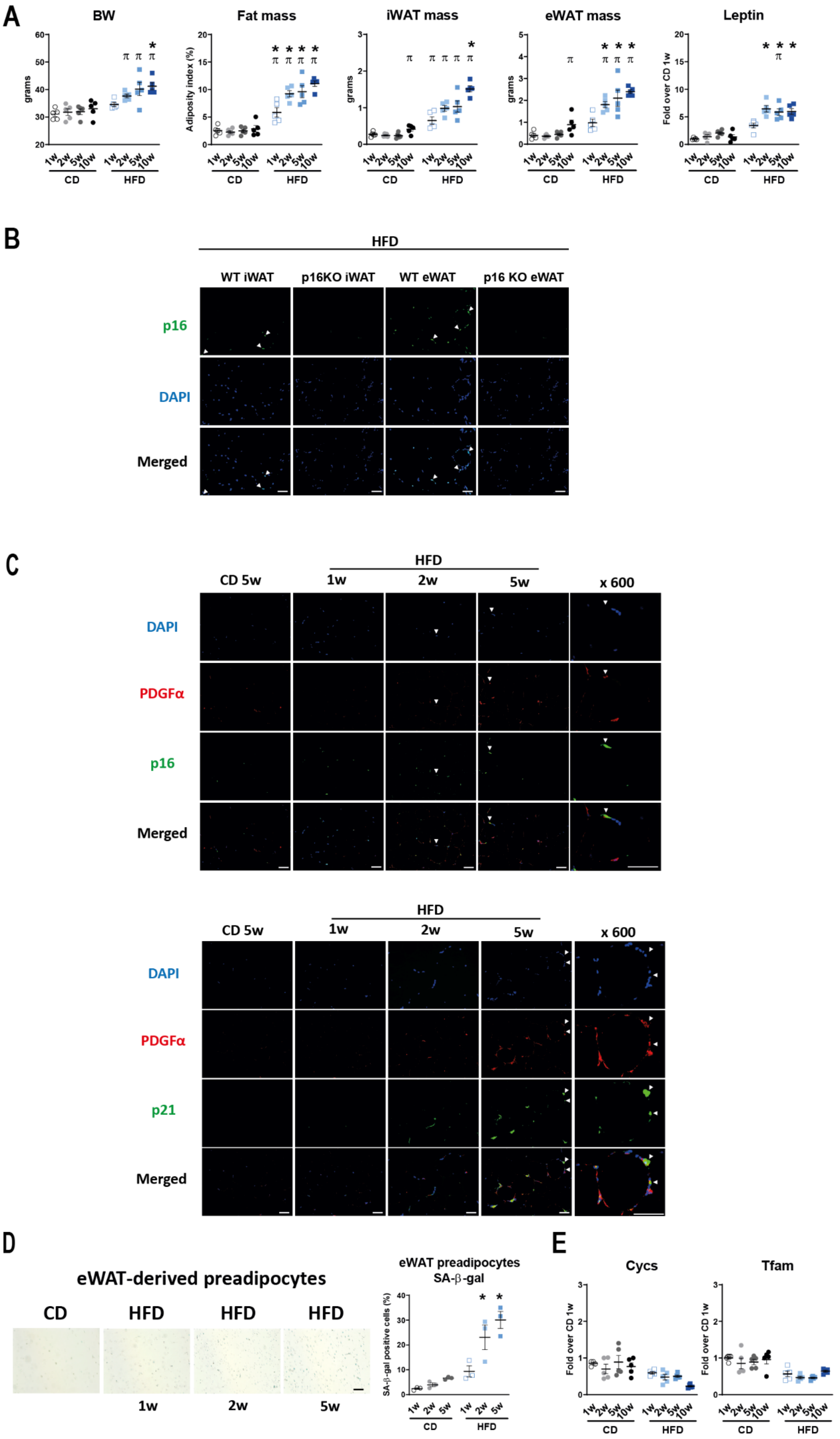
A
BW
Fat mass
iWAT mass
eWAT mass
Leptin
grams
Adiposity index (%)
Fold over CD 1w
1w 2w 5w 10w
CD
HFD
B
HFD
WT iWAT
p16KO iWAT
WT eWAT
p16 KO eWAT
p16
DAPI
Merged
C
CD 5w
HFD
1w
2w
5w
x 600
DAPI
PDGFα
p16
Merged
CD 5w
HFD
1w
2w
5w
x 600
DAPI
PDGFα
p21
Merged
D
eWAT-derived preadipocytes
CD
HFD
HFD
HFD
1w
2w
5w
eWAT preadipocytes
SA-β-gal
SA-β-gal positive cells (%)
E
Cycs
Tfam
Fold over CD 1w

Supplementary Fig. 1. WAT cellular senescence occurs shortly after initiation of HFD. A: Body weight (BW), fat mass (adiposity index), inguinal (iWAT) and epidydimal (eWAT) mass, leptin gene expression in eWAT in a detailed kinetics 1, 2, 5 and 10 weeks after high-fat diet (HFD)- compared with time-matched chow diet (CD)-fed mice; n=5 mice/group. **B:** Testing of p16 antibody in iWAT and eWAT of WT and p16 KO mice as indicated. **C**: Representative immunofluorescence of CD and 1-, 2- and 5-week HFD mice were obtained for PDGFRα (red) and p16 (green, upper panel) or p21 (green, lower panel) counterstained with DAPI (blue) in eWAT; magnification 200x (first four) and x600 (last: 5-week HFD), scale bar=50 µm. **D:** Representative images and quantification of SA–β-gal in preadipocytes derived from eWAT of CD and 1-, 2- and 5-week HFD mice; n=3/group; magnification x200, scale bar=50 µm. **E:** Gene expression of mitochondrial genes (cytochrome C and transcription factor A, Tfam); n=5 mice/group. Data are presented as original images (B-D) or individual values plus mean (A, D-E). Statistical significance was evaluated by one-way ANOVA followed by Bonferroni correction. *$P < 0.05$ vs. CD; $\pi < 0.05$ vs. 1-week HFD.

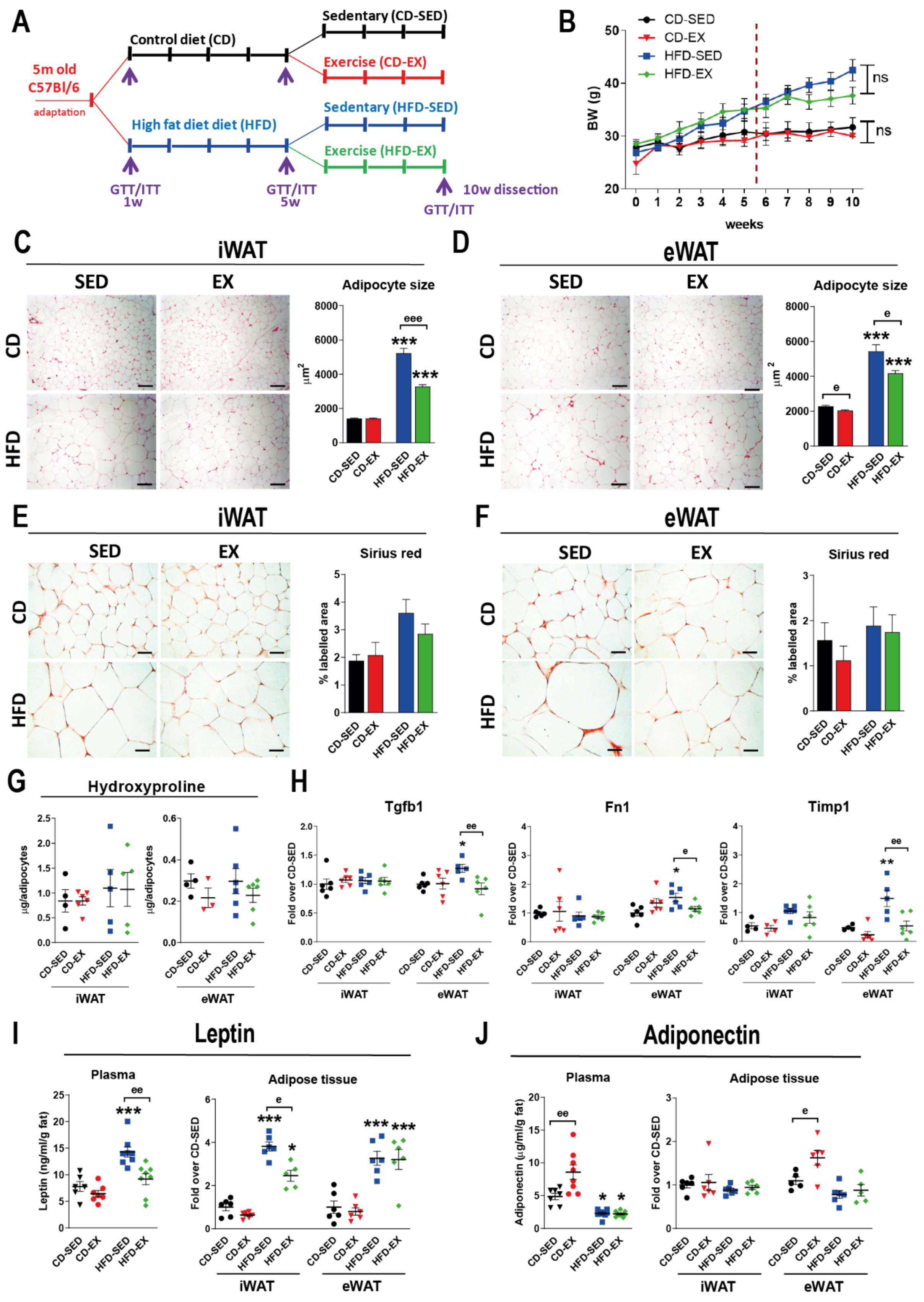
A
5m old C57Bl/6
adaptation
Control diet (CD)
Sedentary (CD-SED)
Exercise (CD-EX)
High fat diet diet (HFD)
Sedentary (HFD-SED)
Exercise (HFD-EX)
GTT/ITT 1w
GTT/ITT 5w
GTT/ITT
10w dissection
B
CD-SED
CD-EX
HFD-SED
HFD-EX
BW (g)
weeks
ns
C
iWAT
SED
EX
CD
HFD
Adipocyte size
D
eWAT
E
iWAT
Sirius red
% labelled area
F
eWAT
G
Hydroxyproline
µg/adipocytes
H
Tgfb1
Fn1
Timp1
Fold over CD-SED
I
Leptin
Plasma
Adipose tissue
Leptin (ng/ml/g fat)
J
Adiponectin
Adiponectin (µg/ml/g fat)

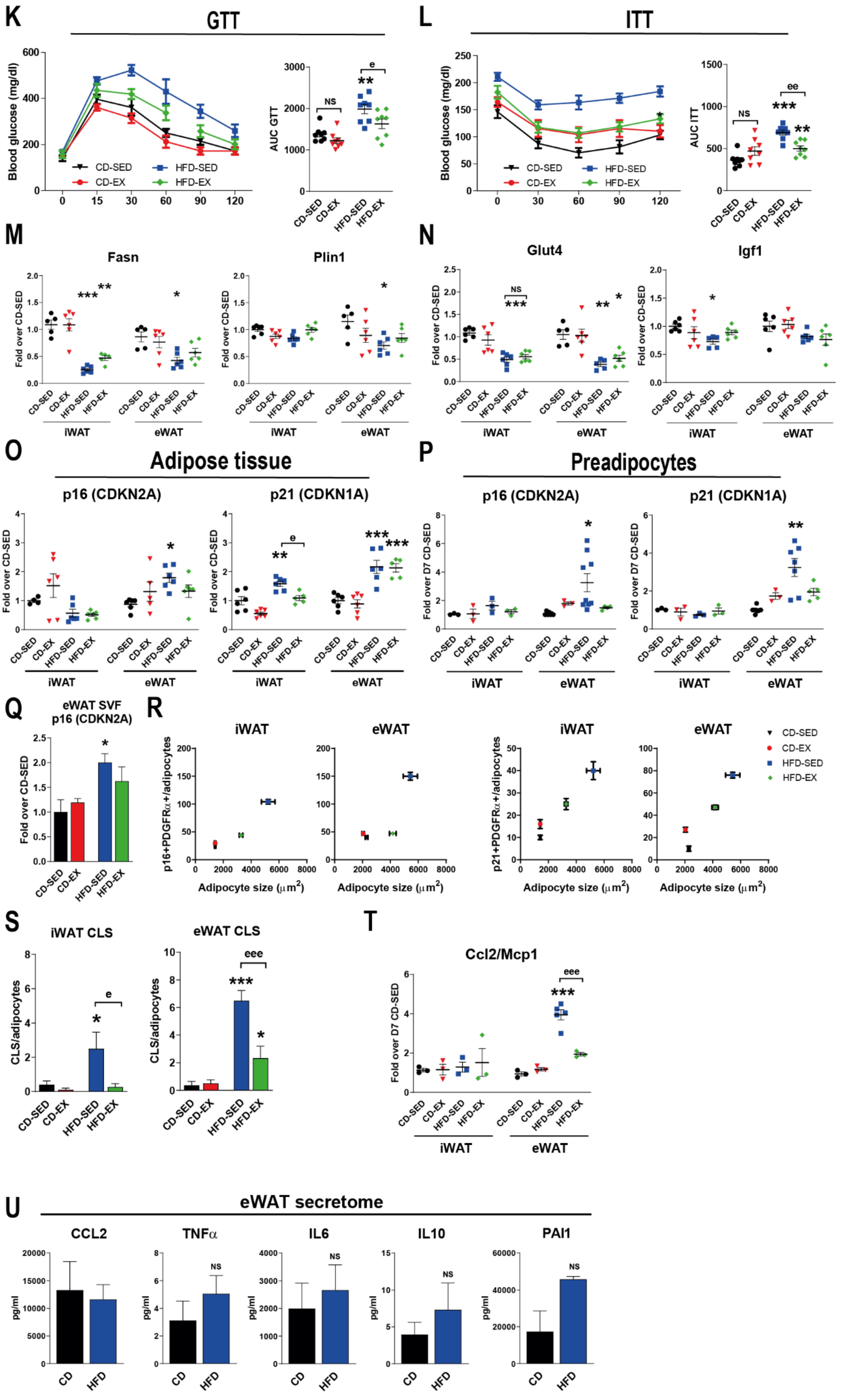
K
GTT
Blood glucose (mg/dl)
CD-SED
CD-EX
HFD-SED
HFD-EX
AUC GTT
NS
e
L
ITT
AUC ITT
ee
M
Fasn
Plin1
Fold over CD-SED
iWAT
eWAT
N
Glut4
Igf1
O
Adipose tissue
p16 (CDKN2A)
p21 (CDKN1A)
P
Preadipocytes
Fold over D7 CD-SED
Q
eWAT SVF
p16 (CDKN2A)
R
p16+PDGFRα+/adipocytes
p21+PDGFRα+/adipocytes
Adipocyte size (μm²)
S
iWAT CLS
eWAT CLS
CLS/adipocytes
eee
T
Ccl2/Mcp1
U
eWAT secretome
CCL2
TNFα
IL6
IL10
PAI1
pg/ml
CD
HFD

Supplementary Fig. 2. HFD induces WAT cellular senescence independent of systemic inflammation. **A**: Experimental design. **B:** Body weight during experimental period; n=8 mice/group. **C-D:** Adipose tissue histology. Representative H&E-stained images of iWAT and eWAT and quantification of adipocyte size; n=5 mice/group; magnification x200, scale bar=50 µm. **E-F:** Sirius red-stained sections of iWAT and eWAT and quantification of fibrotic area expressed in percentage of the total tissue area; n=5 mice/group; magnification x200, scale bar=50 µm. **G:** Hydroxyproline content of iWAT and eWAT, an indicator of total collagen content, normalized by adipocyte number in respective depots; n=6 mice/group. **H:** Quantitative RT-PCR analysis in iWAT and eWAT for key tissue remodeling markers: transforming growth factor beta 1 (Tgfb1), fibronectin 1 (Fn1), tissue inhibitor metalloproteinase 1 (Timp1); n=6 mice/group. **I:** Plasma leptin levels; n=7-8 mice/group. Leptin gene expression in iWAT and eWAT, n=6 mice/group. **J:** Plasma adiponectin levels, n=8 mice/group. Adiponectin gene expression in iWAT and eWAT; n=6 mice/group. **K-L:** Glucose (**K**) and insulin (**L**) tolerance tests with respective areas under the curve (AUC) prior to dissection; n=8 mice/group. **M:** Quantitative RT-PCR analysis of genes involved in both lipogenesis (fatty acid synthase, Fasn) and lipolysis (perilipin 1, Plin1); n=6 mice/group. **N:** Gene expression analysis of glucose (glucose transporter 4, Glut4) and insulin (insulin-like growth factor 1, Igf1) signaling in iWAT and eWAT; n=6 mice/group. **O-P:** Gene expression for p16 and p21 in iWAT and eWAT (**O**) and in iWAT- and eWAT-derived preadipocytes (**P**). For gene expression, results are expressed as fold mRNA change relative to CD-SED values set to 1; n=3-6 mice/group. **Q:** Gene expression for p16 in the stromal vascular fraction (SVF) of the eWAT, day 0, before differentiation; n=3 mice/group. **R:** Biplots showing the association between adipocyte cell size and p16 or p21 expression in the adipocyte precursor pool (p16+PDGFRα+ cells or p21+PDGFRα+) in both WAT depots. **S:** Quantification of crown-like structures (CLS; microscopic foci of dying adipocytes surrounded by macrophages) in iWAT and eWAT, analyzed by immunofluorescence with macrophage specific marker Mac3. CLS quantification was expressed as the mean number over adipocytes; n=5 mice/group. **T:** Quantitative RT-PCR analysis in iWAT and eWAT for monocyte chemoattractant protein-1 (Mcp1/Ccl2), n=6 mice/group. **U:** Epidydimal adipose tissue conditioned media (eWAT secretome) analyzed for a panel of pro-inflammatory cytokines by a Luminex Bio-Plex cytokine assay kit; n=5 mice/group. Data are presented either as original images (C-F), individual values with mean (G-P, T) or mean ± SEM (B-F, K-L, Q-S, U). Statistical significance was evaluated by a one- (C-Q, S-T), two-way (B) ANOVA followed by Bonferroni correction or unpaired, two-tailed t test (U). *P < 0.05; **P < 0.01; ***P < 0.001 for differences due to diet regimen within sedentary and exercise groups (* = diet effect) and ^{e}P < 0.05; ^{ee}P <0.01; ^{eee}P < 0.001 for differences between sedentary and exercise groups fed the same diet (^{e} = exercise effect); ns: nonsignificant.

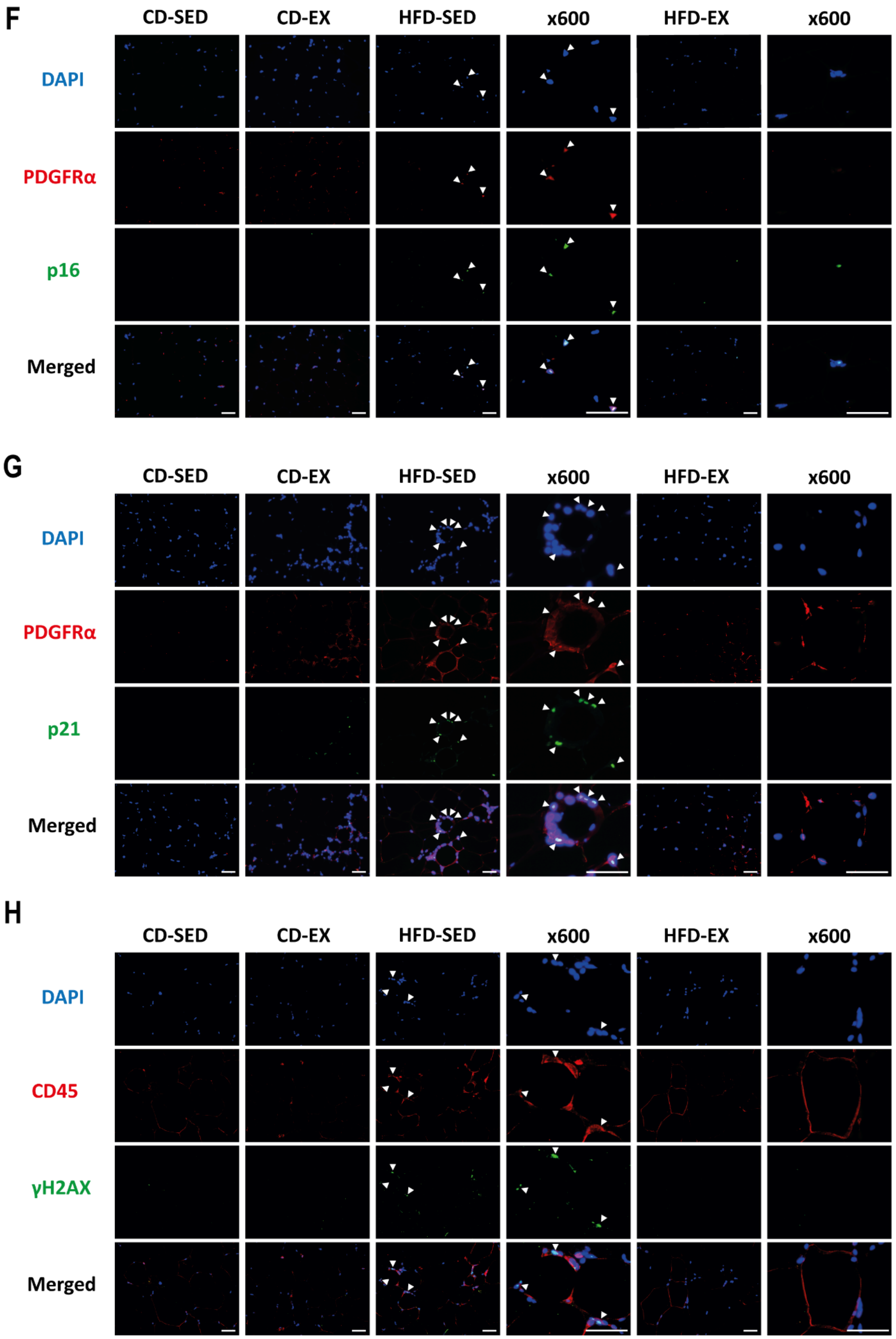
F
CD-SED
CD-EX
HFD-SED
x600
HFD-EX
x600
DAPI
PDGFRα
p16
Merged
G
CD-SED
CD-EX
HFD-SED
x600
HFD-EX
x600
DAPI
PDGFRα
p21
Merged
H
CD-SED
CD-EX
HFD-SED
x600
HFD-EX
x600
DAPI
CD45
γH2AX
Merged

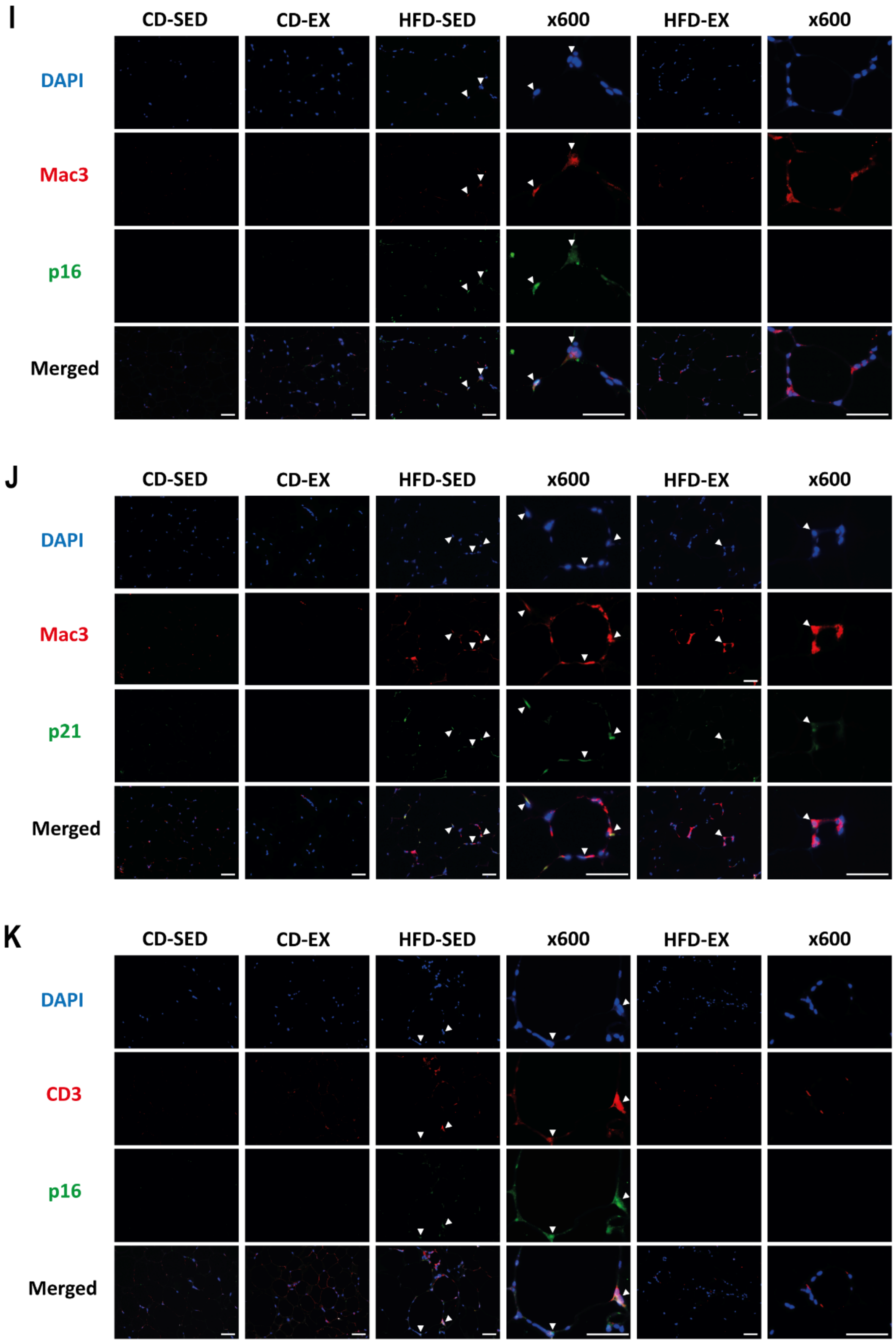

Supplementary Fig. 2*bis*. F-H and I-K: Representative immunofluorescence images of PDGFRα/p16 (red/green; **F**), PDGFRα/p21 (red/green; **G**) and CD45/γH2AX (red/green; **H**) counterstained with DAPI (blue) in eWAT of mice subjected to CD-sedentary (CD-SED), CD-exercise (CD-EX), HFD-sedentary (HFD-SED) and HFD-exercise (HFD-EX) protocols. Magnification x200, unless indicated as x600 magnification for HFD-SED and HFD-EX

images, scale bar=50 μm in all. **I-K**: Representative immunofluorescence images of Mac3/p16 (red/green; **I**), Mac3/p21 (red/green; **J**) and CD3/p16 (red/green; **H**) counterstained with DAPI (blue) in eWAT of mice subjected to CD-sedentary (CD-SED), CD-exercise (CD-EX), HFD-sedentary (HFD-SED) and HFD-exercise (HFD-EX) protocols. Magnification x200, unless indicated as x600 magnification for HFD-SED and HFD-EX images, scale bar=50 μm in all.

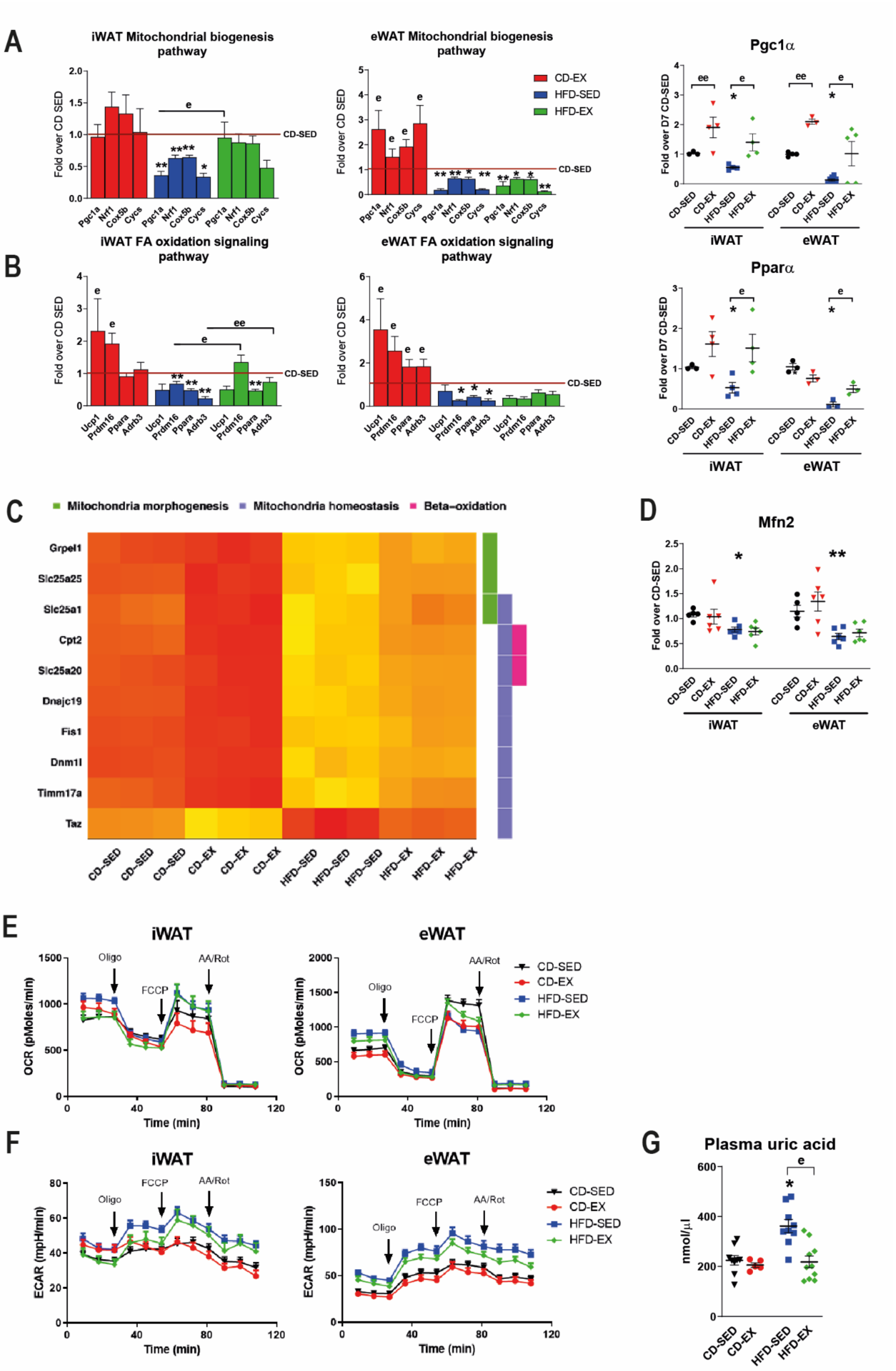
A
iWAT Mitochondrial biogenesis pathway
eWAT Mitochondrial biogenesis pathway
Pgc1α
Fold over CD SED
Fold over D7 CD-SED
CD-SED
CD-EX
HFD-SED
HFD-EX
Pgc1a
Nrf1
Cox5b
Cycs
iWAT
eWAT
B
iWAT FA oxidation signaling pathway
eWAT FA oxidation signaling pathway
Pparα
Ucp1
Prdm16
Ppara
Adrb3
C
Mitochondria morphogenesis
Mitochondria homeostasis
Beta-oxidation
Grpel1
Slc25a25
Slc25a1
Cpt2
Slc25a20
Dnajc19
Fis1
Dnm1l
Timm17a
Taz
D
Mfn2
Fold over CD-SED
E
iWAT
eWAT
OCR (pMoles/min)
Time (min)
Oligo
FCCP
AA/Rot
F
ECAR (mpH/min)
G
Plasma uric acid
nmol/μl

Supplementary Fig. 3. Adipose tissue senescence is associated with increased adipocyte bioenergetics. A: Quantitative RT-PCR analysis in inguinal (iWAT) and epidydimal (eWAT) adipose tissue for mitochondrial biogenesis-related genes: peroxisome proliferator-activated receptor gamma coactivator 1-alpha (PGC-1α), nuclear respiratory factor 1 (Nrf1), cyclooxygenase (Cox)-5b and cytochrome c (Cycs); n=6 mice/group. Gene expression analysis in iWAT- and eWAT-derived preadipocytes for PGC-1α; n=6 mice/group. **B:** Quantitative RT-PCR analysis in iWAT and eWAT for fatty acid oxidation-related genes: uncoupling protein 1 (Ucp1), PR domain containing 16 (Prdm16), peroxisome proliferator-activated receptor alpha (Pparα) and β 3 adrenergic receptor (AdrB3); n=6 mice/group. Gene expression analysis in iWAT- and eWAT-derived preadipocytes for Pparα; n=6 mice/group. **C:** Heatmap showing the expression of a focused panel of genes related to mitochondrial activity. Data obtained from eWAT RNA using RT 2 profiler PCR arrays; n=3 mice/group. **D:** Gene expression analysis in iWAT and eWAT for mitofusin 2 (Mfn2); n=6 mice/group. **E:** Representative oxygen consumption rate (OCR) measured by Seahorse XF24 analyzer. After 3 basal OCR measurements, oligomycin (0.1 μg/ml), FCCP (0.7 μM) and rotenone and antimycin A (Rot and AA, respectively, 1 μM) were injected in sequence; n=4 independent experiments with five technical repeats. **F:** Representative extracellular acidification rate (ECAR) graph, an index of glycolysis, measured by Seahorse XF24 analyzer; n=4 independent experiments with five technical repeats. **G:** Uric acid concentration in plasma; n=5-10 mice/group. Data are presented as individual values with mean (A-B, D, G), as heatmap (C) or mean +/- SEM (A-B, E-F). Statistical significance was evaluated by one- (A-B, D, G) or two-way (E-F) ANOVA followed by Bonferroni correction. *P < 0.05; **P < 0.01; ***P < 0.001 for differences due to diet regimen within sedentary and exercise groups (* = diet effect) and ^{e}P < 0.05; ^{ee}P <0.01; ^{eee}P < 0.001 for differences between sedentary and exercise groups fed the same diet (^{e} = exercise effect).

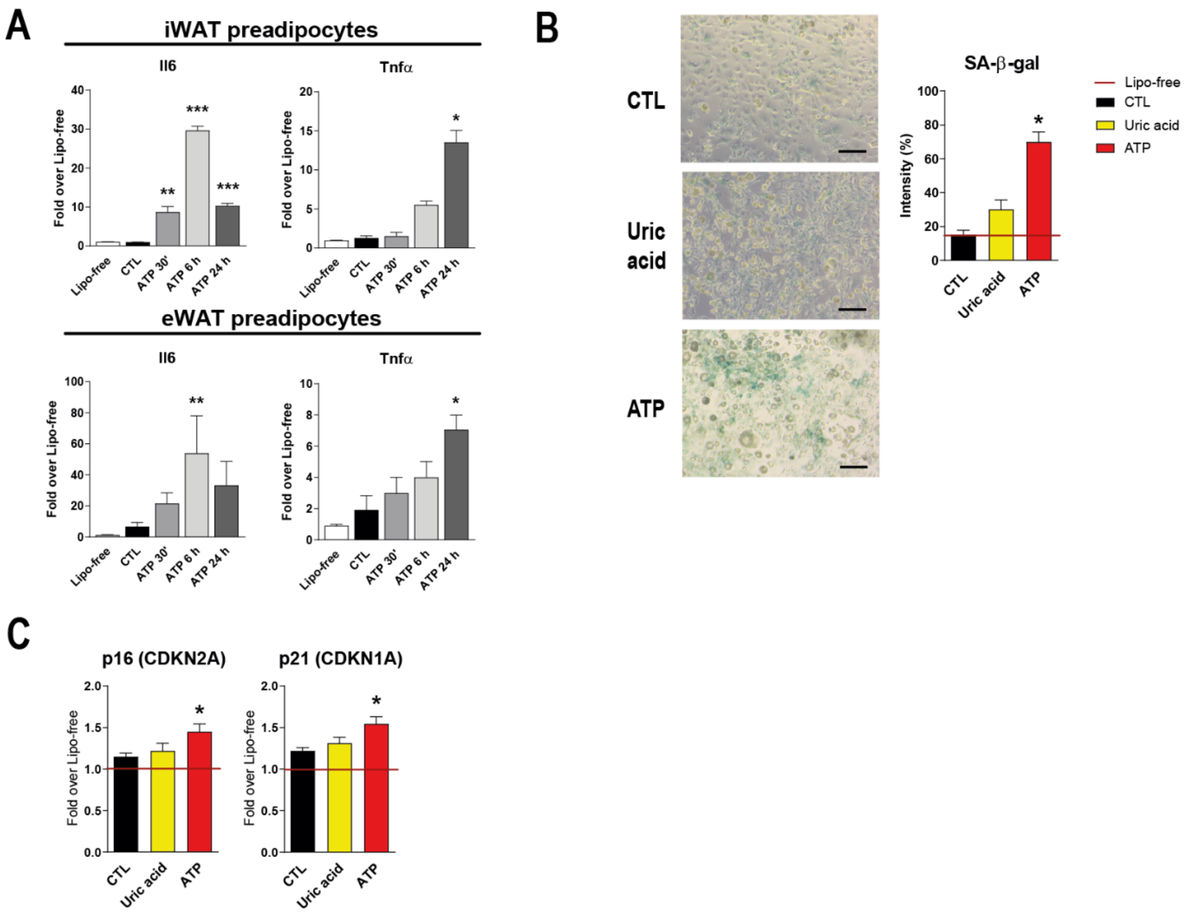

Supplementary Fig. 5. Role of ATP in adipose tissue senescence *in vitro*. A: Gene expression of two SASP markers, IL6 and TNFα, in iWAT- and eWAT-derived preadipocytes over a 24h time course (30' - 6h - 24h) after treatment with ATP-loaded liposomes (200 µM) compared to two control treatments: liposome-free (lipo-free) and liposome alone (CTL). For gene expression, results are expressed as fold change relative to Lipo-free values set to 1; n=3-5 mice/group. **B:** Representative images of β-galactosidase activity (SA–β-gal) in 3T3-L1 cells stimulated for 24h with ATP- and uric acid-loaded liposomes (200 µM) and compared to liposome-free (lipo-free, red line) and liposome alone (CTL); n=3 mice/group, magnification x200, scale bar=50 µm. **C:** Gene expression analysis for senescence (p16, p21) in 3T3-L1 cells stimulated for 24h with ATP- and uric acid-loaded liposomes with liposome-free (lipo-free, red line) and liposome alone (CTL); n=3-6 mice/group. Data are presented as original images (B) or mean +/- SEM (A-C). Statistical significance was evaluated by one-way ANOVA followed by Bonferroni correction. $*P < 0.05$; $**P < 0.01$; $***P < 0.001$ for differences due to treatment compared to liposome alone (CTL).

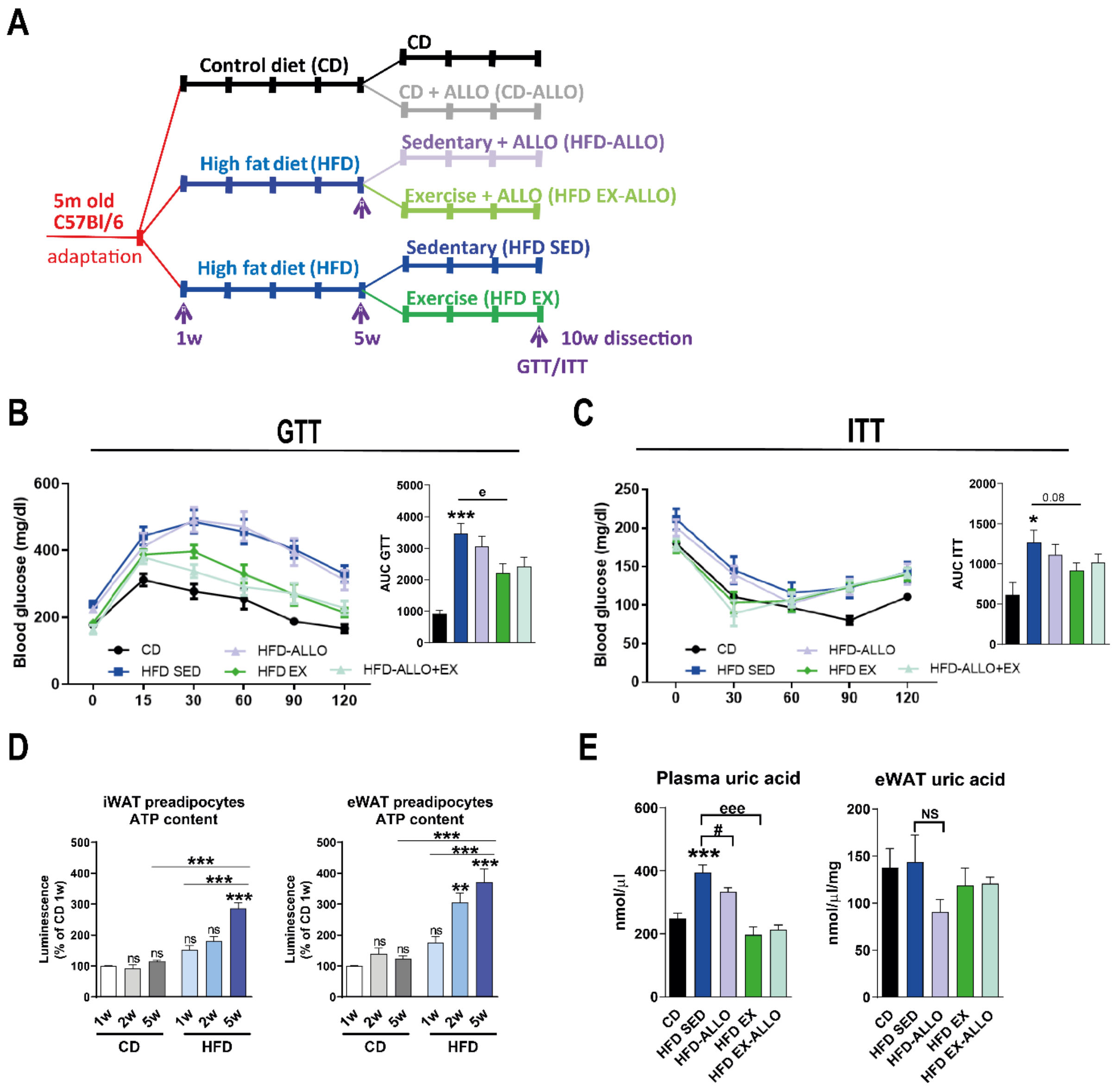

Supplementary Fig. 6. Role of ATP in adipose tissue senescence *in vivo*. A: Experimental design. **B-C:** Glucose (B) and insulin tolerance test (C) pre-dissection with respective areas under the curve (AUC); n=5-10 mice/group. **D:** Intracellular ATP levels in preadipocytes derived from iWAT or eWAT in mice fed CD or HFD for indicated periods; n=3-8 mice per group. **E:** Uric acid levels in plasma and eWAT lysates; n=4-7 mice/group. All data are presented as mean ± SEM. *P < 0.05; **P < 0.01; ***P < 0.001 for differences due to diet regimen within sedentary and exercise groups (* = diet effect); ^{e}P < 0.05; ^{ee}P <0.01; ^{eee}P < 0.001 for differences between sedentary and exercise groups fed the same diet (^{e} = exercise effect); ns: nonsignificant.

F

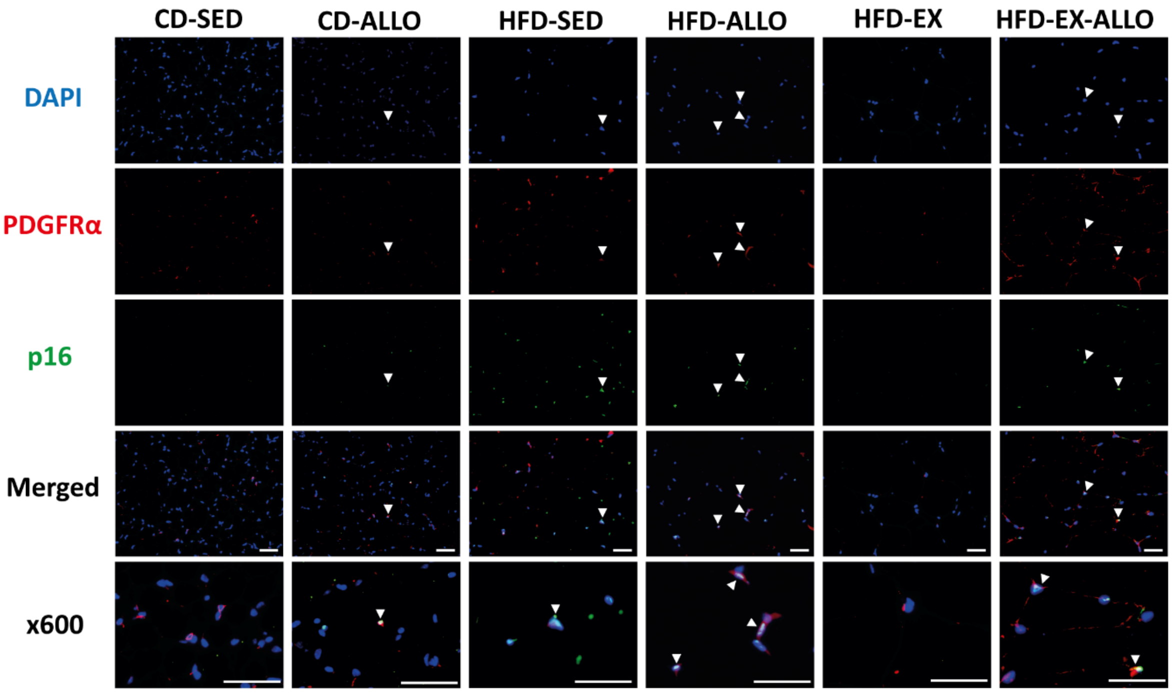

G

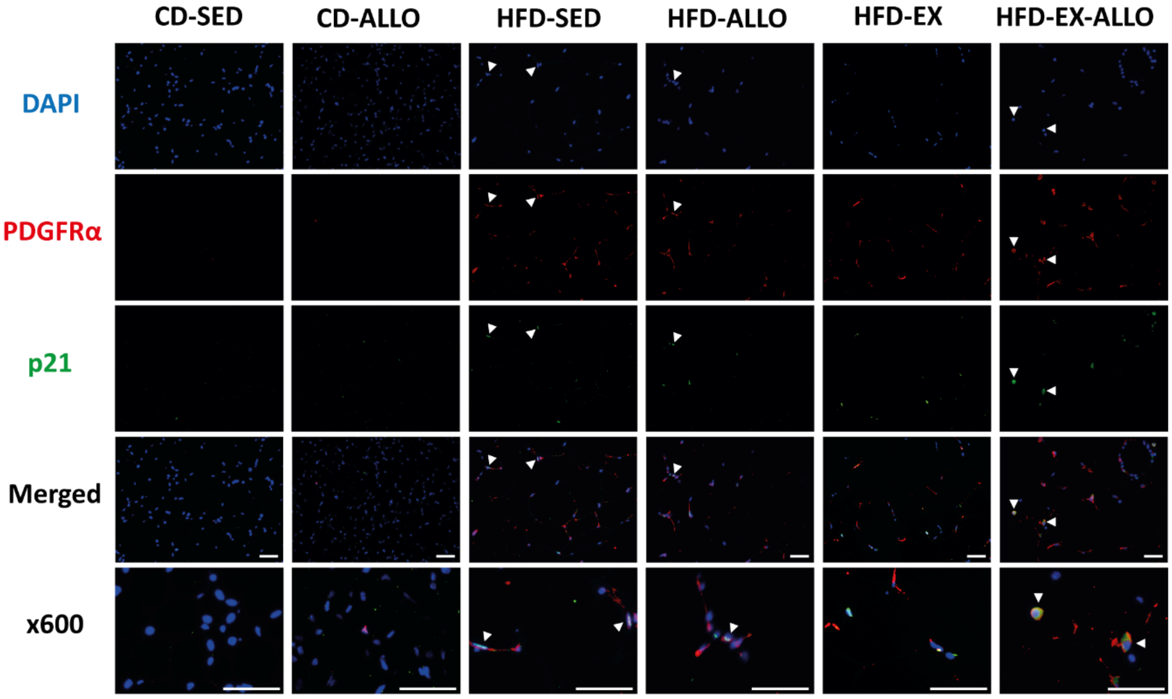

Supplementary Fig. 6*bis*: iWAT. *F-G:* Representative immunofluorescence images of PDGFRα/p16 (red/green; **F**) and PDGFRα/p21 (red/green; **G**) counterstained with DAPI (blue) in iWAT of mice subjected to CD-sedentary (CD-SED), CD-exercise (CD-EX), HFD-sedentary (HFD-SED), HFD-SED treated with allopurinol (HFD-ALLO), HFD-exercise (HFD-EX) and HFD-exercise treated with allopurinol (HFD EX-ALLO) protocols. Magnification x200, except in the lowest row where it is x600, scale bar=50 μm in all.

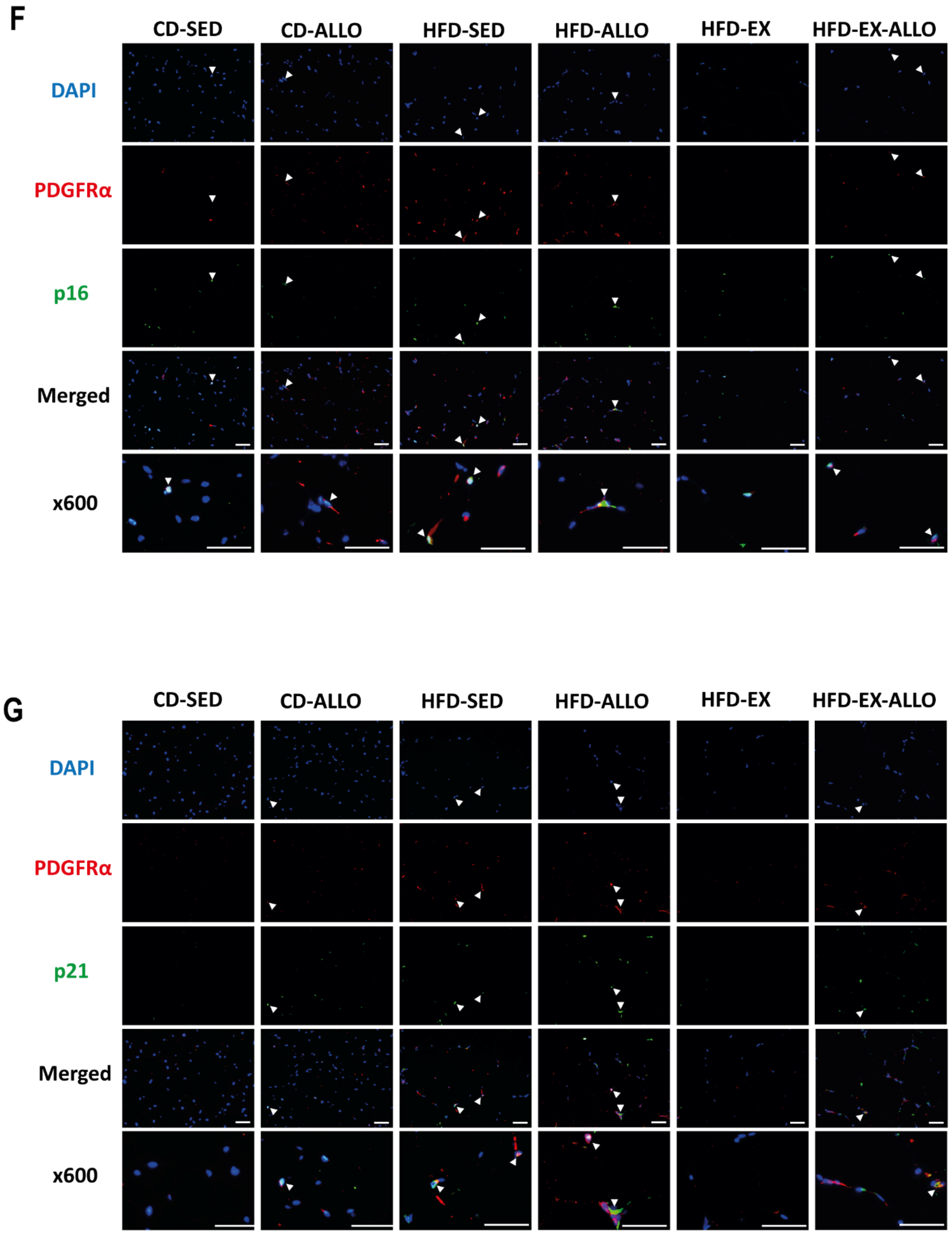

Supplementary Fig. 6*bis*: eWAT. *F-G:* Representative immunofluorescence images of PDGFRα/p16 (red/green; **F**) and PDGFRα/p21 (red/green; **G**) counterstained with DAPI (blue) in eWAT of mice subjected to CD-sedentary (CD-SED), CD-exercise (CD-EX), HFD-sedentary (HFD-SED), HFD-SED treated with allopurinol (HFD-ALLO), HFD-exercise (HFD-EX) and HFD-exercise treated with allopurinol (HFD EX-ALLO) protocols. Magnification x200, except in the lowest row where it is x600, scale bar=50 µm in all.

Supplementary methods

Exercise training

The exercise consisted of 2 daily swimming sessions (up to 60 minutes in the morning and 30 minutes in the afternoon) separated by a 6 hour's break (Derumeaux et al, 2008). Transparent Plexiglas tanks were filled with tap water, maintained at ambient temperature (31 ± 1°C), with the latter monitored by a floating glass mercury thermometer. The first exercise week was used for aquatic training with low water level from the bottom. From the second week, water level was raised to ensure that mice did not touch the bottom. Swimming sessions were supervised to avoid floating and/or clinging of individual animals. The duration was increased by 10 minutes each day, until reaching 90 min/day for 5 days/week, for a total of 4 weeks. To maintain mobility, small waves were caused without disturbing the mice. After training, mice were gently dried using paper towels and left in their cage under a heating lamp to prevent hypothermia until they were completely dried.

Hydroxyproline assay

Hydroxyproline measurement was performed in frozen powdered samples of iWAT and eWAT using a colorimetric assay kit (BioVision, Inc, Milpitas, CA, USA) (Marcelin et al. 2017).

In Vivo Bioluminescent Imaging details

Isoflurane-anesthetized p16^{LUC} heterozygote mice were injected intraperitoneally with D-luciferin potassium salt (15 mg/mL in PBS; PerkinElmer) and imaged using PhotonIMAGER Optima (Biospace Lab, Nesles la Valée, France). Bioluminescence (BLI) was calculated as indicated in the formula (BLI = BLI ROI area of interest (in Ph/s/sr) / BLI ROI

LED (internal control –LED- in ph/s/sr), where Ph/s/sr is photon per second per steradian. Areas of interest analyzed: abdominal cavity and total body.

Estimation of mitochondrial mass by ImageJ

The image analysis of Tom20 staining was performed in a blinded manner by the same operator. Images specifically selected to avoid crown-like structures (that feature cells with high mitochondrial content and hence would act as a confounder when compared to adipocytes with low mitochondrial content) were processed to adjust the brightness and contrast using the automated routine of the program. The positive signal was processed as "binary" and particle intensity was measured. Particle intensity was subsequently normalized to adipocyte count."

Mitochondrial enzymatic activities

Frozen mature adipocytes and eWAT tissue were permeabilized in Extraction Buffer (20 mM Tris-HCl, 250 mM sucrose, 2 mM EGTA, 40 mM KCl and 1 mg/mL BSA, pH 7.2) with Percoll and 100 µg/mL digitonin. After centrifugation at 2300 g and 10 000 g, respectively, sample pellet was collected for enzymatic activity measurements.

<u>Citrate synthase enzymatic activity</u> was measured in real-time after addition of 150 µM 5,5'-Dithio-bis 2-nitrobenzoic acid, 300 µM acetyl-coA and 1 mM oxaloacetate in Tris 10 mM, pH 8 in 96-well microplates by spectrofluorimeter (Tecan Infinite 200; OD at 415 nm).

<u>Cytochrome c oxidase enzymatic activity</u> was measured in real-time after addition of 100 µM reduced bovine heart cytochrome c in KH_2PO_4 10 mM in 96-well microplates by spectrofluorimetry (Tecan Spark; OD at 550 nm). KCN 300 µM was used as reference inhibitor.

ATP synthase activity was measured in real-time after addition of 2 mM ATP, 25 mM phosphoenolpyruvate, 7.5 U pyruvate kinase, 12.5 U lactate dehydrogenase, 500 µM NADH, 1 µM antimycin A in reaction buffer (KH_2PO_4 25 mM, sucrose 250 mM, KCl 30 mM, EGTA 0.2 mM, $MgCl_2$ 5 mM, P1,P5-Di(adenosine-5′) pentaphosphate pentasodium salt (Ap5A) 2 µM, BSA 0.1%) in 96-well microplates by spectrofluorimetry (Tecan Spark; OD at 340 nm). Oligomycin A 10 µM was used as reference inhibitor.

Enzymatic activity was normalized by protein quantity of the cell extracts, assessed by BCA assay. All reagents were purchased from Sigma Aldrich (Saint-Quentin-Fallavier, France).

Adipose tissue explants

Paired iWAT and eWAT depots were collected and kept at room temperature in a 24-well plate with 1 ml DMEM/well. Fat tissue (0.1 g) was minced and incubated for 1 hour at 37°C and 5% CO2 prior to transfer into a new plate with freshly prepared transfer medium (DMEM with 4,5 g glucose and glutamine containing 1% free fatty acid bovine serum albumin and 1% antibiotic and antimycotic solution). The conditioned medium was collected 24 hours after incubation and stored at –80°C until analysis of secretome.

3T3-L1 culture

Murine 3T3-L1 preadipocytes were cultured using DMEM containing 10% newborn calf serum and antibiotics. For differentiation, cells were stimulated with 3T3-L1 differentiation medium containing IBMX (500 µM), dexamethasone (250 nM), and insulin (175 nM) for 2 days after cells reached confluence. Then, the medium was changed for DMEM containing 10% FBS and insulin (175 nM) and adipocytes were then kept in DMEM containing only 10% FBS.

Supplementary tables

Supplementary table 1:

Table 1. Morphometric parameters of mice selected and used for bioenergetic, histological and molecular analysis of adipose tissue function (iWAT and eWAT).

	CD SED	HFD SED	CD EX	HFD EX
Body composition				
Body weight (g)	32.4 ± 1.8	45.0 ± 5.7*	30.2 ± 1.8	40.7 ± 4.9*
Adiposity index (%)	3.6 ± 0.8	12.6 ± 1.6*	4.1 ± 1.3	10.6 ± 2.2*
Organ weight				
iWAT (g)	0.37 ± 0.03	2.02 ± 0.20*	0.43 ± 0.04	1.35 ± 0.23*
eWAT (g)	0.63 ± 0.05	2.81 ± 0.18*	0.64 ± 0.09	2.27 ± 0.17*
prWAT (g)	0.17 ± 0.03	0.91 ± 0.14*	0.18 ± 0.03	0.77 ± 0.10*

Data are expressed as means ± SEM (n=8 mice/group); Adiposity index represents the ratio of white adipose depots, inguinal (iWAT) + epidydimal (eWAT) + perirenal (prWAT) fat pad weights over body weight. Statistical significance was evaluated by one-way ANOVA followed by Bonferroni correction. *: $p<0.05$ vs. CD SED mice.

Supplementary table 2:

Antibodies and Taqman assay IDs

Target protein	Clone/product number	company
**p16**	2D9A12/ab54210	Abcam, UK
**p21**	EPR18021/ab188224	Abcam, UK
**p21**	C-19/sc-397-G	Santa Cruz, U.S.A.
**PDGFRα**	BAF1062	R&D System, U.S.A.
**PDGFRα**	EPR22059-270/ab203491	Abcam, UK
**Mac3**	CD107b/ab550292	BD Biosciences
**CD3**	ab5690	Abcam, UK
**CD68**	ab125212	Abcam, UK
**CD45**	AB550539	BD Pharmingen
**Anti-gamma H2A.X (phospho S139)**	EP854(2)Y/ab81299	Abcam, UK
**Oxoguanine 8**	2Q2311/ ab206461	Abcam, UK
**Perilipin**	k-13/ sc-47320	Santa Cruz, U.S.A.
**Tom20**	FL-145/sc-11415	Santa Cruz, U.S.A.
**Xanthine Oxidase**	EPR4605/ab109235	Abcam, UK

Target gene	Taqman assay ID
**Adipoq**	Mm00456425_m1
**Adrb3**	Mm02601819_g1
**ATP5a1**	Mm00431960_m1
**Cdkn1a**	Mm04205640_g1
**Cdkn2a**	Custom primer for transcript NM_00104065.1
**Col1a**	Mm00801666_g1
**Cox5b (Gm11273)**	Mm01229713_g1

**Ctgf**	Mm01192933_g1
**Cycs**	Mm01621048_s1
**Fasn**	Mm00662319_m1
**Fn1**	Mm01256744_m1
**Glb1**	Mm00515342_m1
**Hif1a**	Mm00468869_m1
**Igf1**	Mm00439560_m1
**Il6**	Mm00446190_m1
**Lep**	Mm00434759_m1
**Lipe/Hsl**	Mm00495359_m1
**Lox**	Mm00495386_m1
**Mcp1/Ccl2**	Mm00441242_m1
**Mfn2**	Mm00500120_m1
**Nrf1**	Mm01135606_m1
**Pai1/Serpine1**	Mm00435858_m1
**PdgfRa**	Mm00440701_m1
**Plin1**	Mm00558672_m1
**Pnpla2**	Mm00503040_m1
**Ppara**	Mm00440939_m1
**Ppargc1α**	Mm01208835_m1
**Prdm16**	Mm00712556_m1
**P2rx7**	Mm01199500_m1
**Slc2a4**	Mm00436615_m1
**Tgfb1**	Mm01178820_m1
**Timp1**	Mm01341361_m1
**Tnf**	Mm00443258_m1

**Trp53**	Mm01731290_g1
**Ucp1**	Mm01244861_m1

Supplementary table 3: Details on staining: markers, sample type, number of samples and replicates (for histology, sections per each mouse; for cell culture, different well per each condition).

**MAIN FIGURES**	**Markers**	**Sample type**	**Number of samples**	**Number of replicates**
Fig. 1A	SA-β-gal	eWAT	5/group	none
Fig. 1C	PDGFRα+p16+	eWAT	3/group	1-5/mouse
Fig. 1C	PDGFRα+p21+	eWAT	3-4/group	2-6/mouse
Fig. 2B-C	SA-β-gal	iWAT-eWAT	5-7/group	none
Fig. 2D-E	SA-β-gal	iWAT-eWAT preadipocytes	4/group	2/well
Fig. 2F	PDGFRα+p16+	iWAT-eWAT	4-5/group	5-6/mouse
Fig. 2G	PDGFRα+p21+	iWAT-eWAT	4/group	3-5/mouse
Fig. 2H	γH2AX+	eWAT	3/group	4/mouse
Fig. 2I	Mac3+ p16+	iWAT-eWAT	3-4/group	3-5/mouse
Fig. 2J	Mac3+ p21+	iWAT-eWAT	3/group	4-5/mouse
Fig. 2K	CD3+ p16+	iWAT-eWAT	3-5/group	4/mouse
Fig. 3A	Tom20	iWAT-eWAT	3-7/group	3-5/mouse
Fig. 3H	XO	iWAT-eWAT	3/group	3/mouse

Fig. 3I	XO	iWAT-eWAT preadipocytes	3/group	none
Fig. 5F-G	SA-β-gal	iWAT-eWAT preadipocytes	4-5/group	2/well
Fig. 6B	SA-β-gal	iWAT-eWAT	5/group	none
Fig. 6D-E	PDGFRα+p16+	iWAT-eWAT	3/group	3-6/mouse
Fig. 6F-G	PDGFRα+p21+	iWAT-eWAT	3-5/group	3-6/mouse
**SUPPLEMENTARY FIGURES**				
Fig. S1C	SA-β-gal	iWAT-eWAT preadipocytes	3/group	3/mouse
Fig. S2C-D	H&E	iWAT-eWAT	5/group	3/mouse, >200 adipocytes/per mouse
Fig. S2E-F	Sirius-red	iWAT-eWAT	5/group	3/mouse
Fig. S2S	Crown-like structures (Mac3+)	iWAT-eWAT	5/group	3/mouse
Fig. S5B	SA-β-gal	3T3-L1	3/group	2/well
Fig. S5D	p21+	iWAT-eWAT preadipocytes	3/group	2/well
